# Supplementary material for: Readily Available Water Access is Associated with Greater Milk Production in Grazing Dairy Herds
Source: Animals (Basel). 2019 Feb 5;9(2):48. doi: 10.3390/ani9020048 (PMC6406619; doi:10.3390/ani9020048)
Supplement: Supplementary file 1 [file animals-09-00048-s001.zip › animals-433944-SI.pdf]

Data analyis for Readily available water access is associated with greater milk production in grazing dairy herds

by Ruan Daros

Dec 4, 2018

Contents

1 Introduction 1
1.1 Objective . . . 2

2 Statistical analysis 2
2.1 Data screening . . . 2
2.1.1 **Outcome variable** . . . 2
2.1.2 **Predictor of interest** . . . 3
2.1.3 Descriptive variables . . . 3
2.1.3.1 Continuous variables . . . 4
2.1.3.2 Categorical variables . . . 14
2.1.4 Unconditional modelling . . . 16
2.1.5 Hypothesis testing - Multivariable analysis . . . 37
2.1.5.1 Selected variables . . . 37
2.1.5.2 Final model . . . 38

3 Conclusion 41

1 Introduction

The data herein used is part of a larger project set out to investigate the prevalence and risk factors for lameness and transition period diseases in dairy cows under intensive pasture based systems. Data presented in this study were collected by Ruan Daros and Jose Bran from February to October of 2015 in 53 dairy herds in the west of Santa Catarina state, Brazil. More details are provided in Daros et al., (2017) and Bran et al., (2018).

The research team for the overarching project:

1. Maria J. Hotzel - Universidade Federal de Santa Catarina (principal investigator - Brazil)
2. Marina A. G. von Keyserlingk - University of British Columbia (principal investigator - Canada)
3. Jose Bran - Universidade Federal de Santa Catarina (PhD student - Brazil)
4. Ruan Daros - University of British Columbia (PhD student - Canada)
5. Stephen LeBlanc - University of Guelph (Collaborator - Canada)
6. Gabriela Olmos - Universidade Federal de Santa Catarina (Post-Doc - Brazil)
7. Alexi Thompson - University of British Columbia (Msc student - Canada)
8. Melissa Garcia - Universidade de Antioquia (undergrad student - Colombia)
9. Davi Rios Lopez - Universidade Nacional de Colombia (undergrad student - Colombia)
10. Guilherme Rodrigues - Universidade Federal de Santa Catarina (undergrad student - Brazil)

This document contains the statistical procedures used to analyze the data for the paper: **Readily available water access is associated with greater milk production in grazing dairy herds**; by Daros et al., XXXX.

1.1 Objective

- To measure the association between herd milk yield and different types of water provision for grazing dairy cows

2 Statistical analysis

Data for this project contains several hundreds of variables and confidential information. Thus, data were anonymized and only variables of interest to address the objective of the study were kept. The data set used in this analisys is available as a companion file.

2.0.0.0.1 Packages used

```
library(tidyr)
library(dplyr)
library(car)
library(ggplot2)
library(ggpubr)
```

2.1 Data screening

2.1.1 Outcome variable

Average milk yield per cow was calculated using daily average of the bulk tank milk from the last days reported by farmer and cross referenced with milk shipment receipts/records (referent to the time of the first visit) divided by the number of lactating cows at the visit day.

```
hist(DF$avg.cow.d, main = "Distribution of farm average milk yield - kg/d.cow",
     xlab = "kg of milk per cow per day")
```

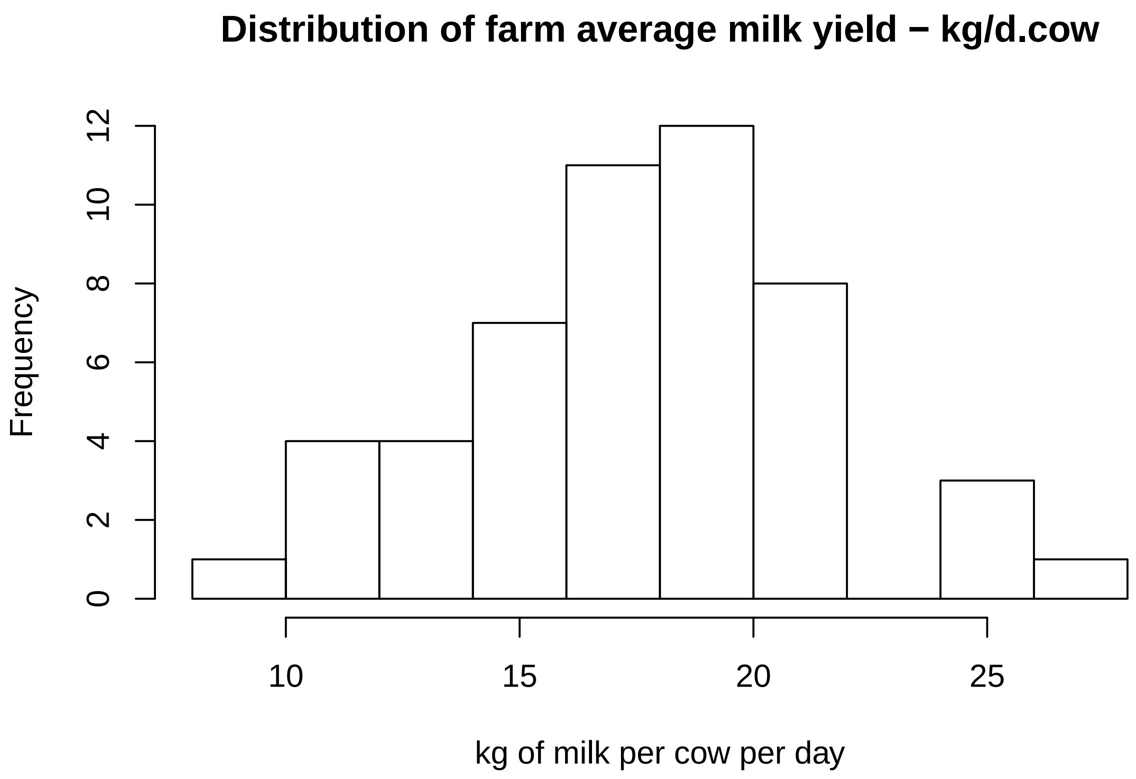


```
summary(DF$avg.cow.d)
```

```
##    Min. 1st Qu.  Median    Mean 3rd Qu.    Max.    NA's 
##   8.654  14.816  17.812  17.689  20.000  27.083       2
```

```
sd(DF$avg.cow.d, na.rm = T)
```

```
## [1] 3.987827
```

NAs: Two farms did not have their daily milk average data collected.

2.1.2 Predictor of interest

Restricted (FREE) - cows did not have access to a water trough while on pasture and 2) Unrestricted (LIMITED) - cows had free access to a water trough while on pasture.

```
table(DF$water.access)
```

```
## 
##    free limited 
##      27      25
```

2.1.3 Descriptive variables

Herd size

Number of milking cows. Dry cows and heifers not included.

```
summary(DF$n.milk.cows)
```

```
##    Min. 1st Qu.  Median    Mean 3rd Qu.    Max.    NA's 
##   22.00   30.00   34.50   36.92   41.25   67.00       1
```

```r
sd(DF$n.milk.cows, na.rm =T)
```

```
## [1] 9.909089
```

Number of fresh paddocks per day

```r
table(DF$hours.paddock)
```

```
##
##  8 12 24
##  1 43  8
```

8h/paddock = 3 paddocks a day, 12h/paddock = 2 paddocks a day and 24h/paddock = 1 paddock per day. We will explore if this variable has an effect on milk production in the sections below.

Paddock type: In rotational grazing systems paddocks can have fixed size - i.e. paddocks are built accordingly to herd size and average pasture production - or variable, which means that the farmer decides the size of the paddocks accordingly to the amount of pasture available.

```r
table(DF$paddock.type)
```

```
##
##     both    fixed variable
##        6       26       20
```

2.1.3.1 Continuous variables

Number of lactations:

```r
hist(DF$mean_lac, main = "Distribution of mean parity per farm",
     xlab = "parity")
```

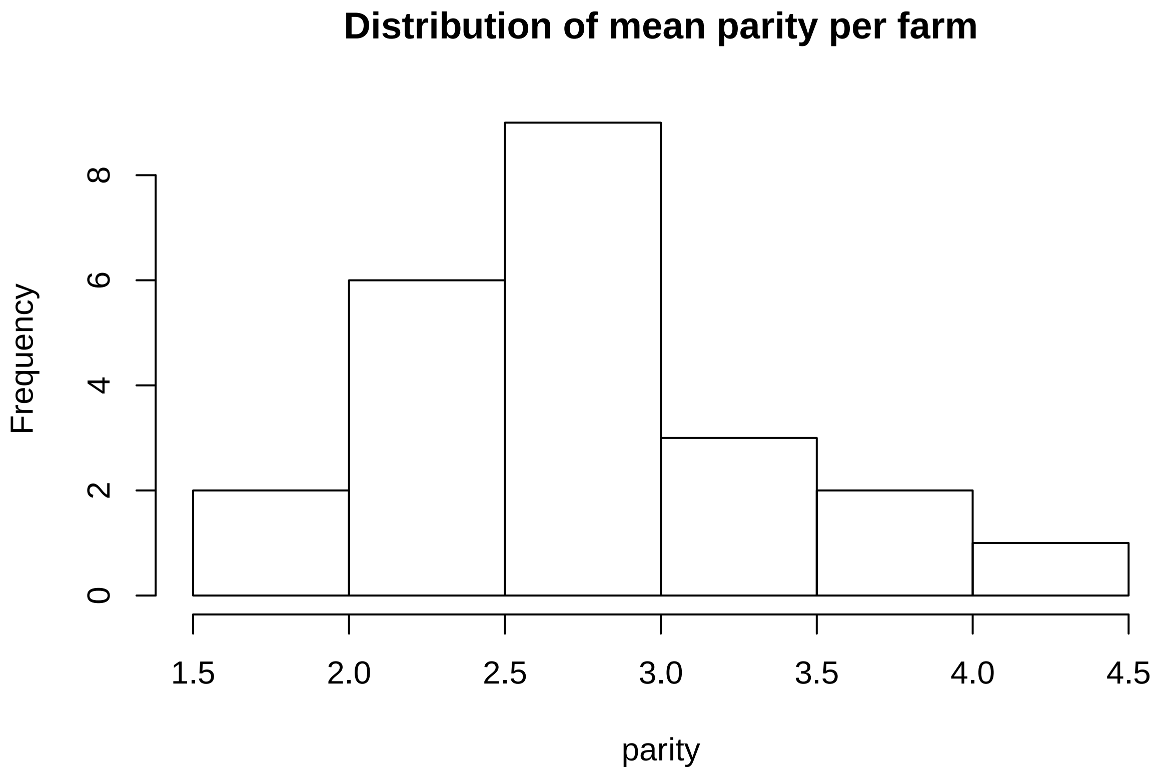

```r
summary(DF$mean_lac)
```

```
##    Min. 1st Qu.  Median    Mean 3rd Qu.    Max.    NA's 
##   1.536   2.270   2.676   2.731   3.111   4.250      30
```

```r
summary(lm(mean_lac ~ water.access, data = DF))
```

```
## 
## Call:
## lm(formula = mean_lac ~ water.access, data = DF)
## 
## Residuals:
##     Min      1Q  Median      3Q     Max 
## -1.0336 -0.3735 -0.1145  0.4567  1.3888 
## 
## Coefficients:
##                     Estimate Std. Error t value Pr(>|t|)    
## (Intercept)           2.8612     0.1812  15.793 9.27e-13 ***
## water.accesslimited  -0.3549     0.2687  -1.321    0.202    
## ---
## Signif. codes:  0 '***' 0.001 '**' 0.01 '*' 0.05 '.' 0.1 ' ' 1
## 
## Residual standard error: 0.6276 on 20 degrees of freedom
##   (31 observations deleted due to missingness)
## Multiple R-squared:  0.08021,    Adjusted R-squared:  0.03422 
## F-statistic: 1.744 on 1 and 20 DF,  p-value: 0.2015
```

Unfortunately we have not collected enough data regarding number of lactations. However, given the data we have (~20 herds), there is no evidence that farms that provided unrestricted access to water have different mean_lac than farms that restricted water access to their cows. Due to this variable containing to many missing values and no evidence that mean_lac is unbalcend between water access types we will not be consider mean_lac for modelling.

Breed: as continuous; % percentage of Holsteins cows in the lactating herd at on the visit day.

```r
hist(DF$holpercent, main = "Distribution of % of
  holstein cows per farm",
     xlab = "% of holstein cows")
```

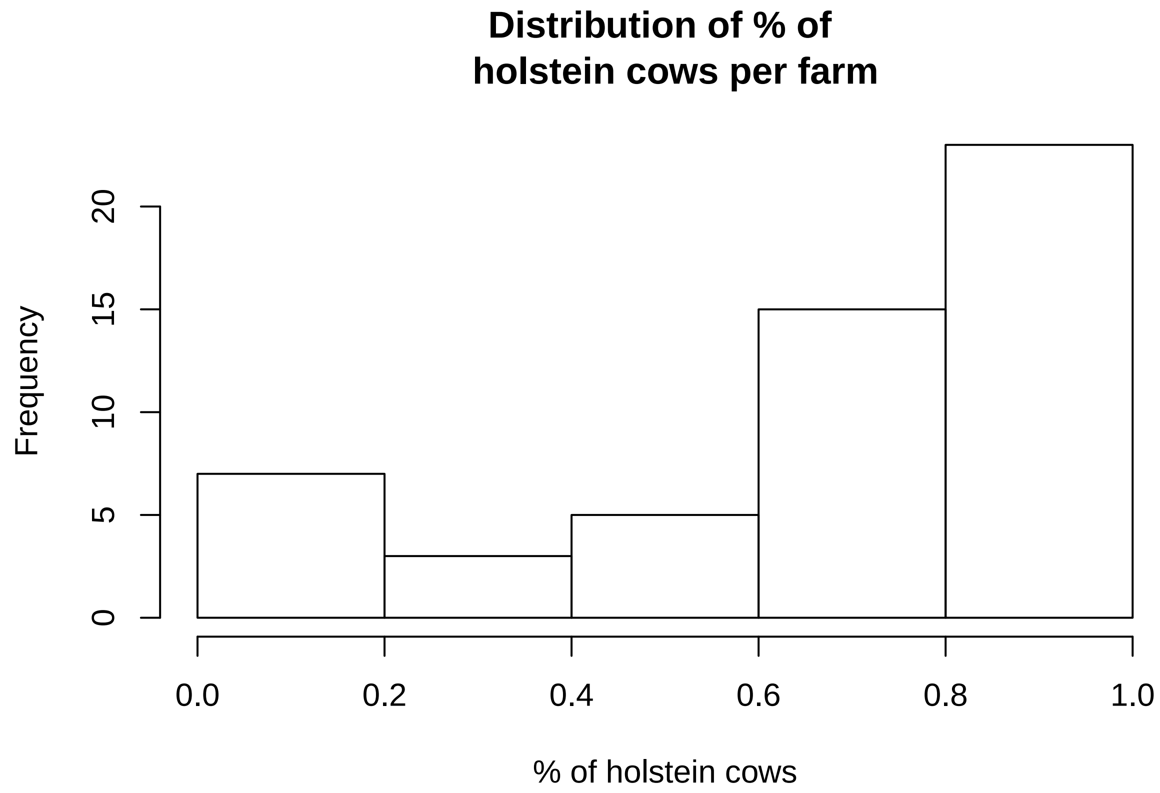


```
summary(DF$holpercent)
```

```
##    Min. 1st Qu.  Median    Mean 3rd Qu.    Max.
##  0.0000  0.5278  0.7500  0.6837  0.9688  1.0000
```

Average amount of concentrate: Offered per cow per day, as reported by the farmer.

```
hist(DF$concentrate.kg.cow.d, main = "Distribution of Kg of concentrate
     offered per cow/d",
     xlab = "kg of concentrate")
```

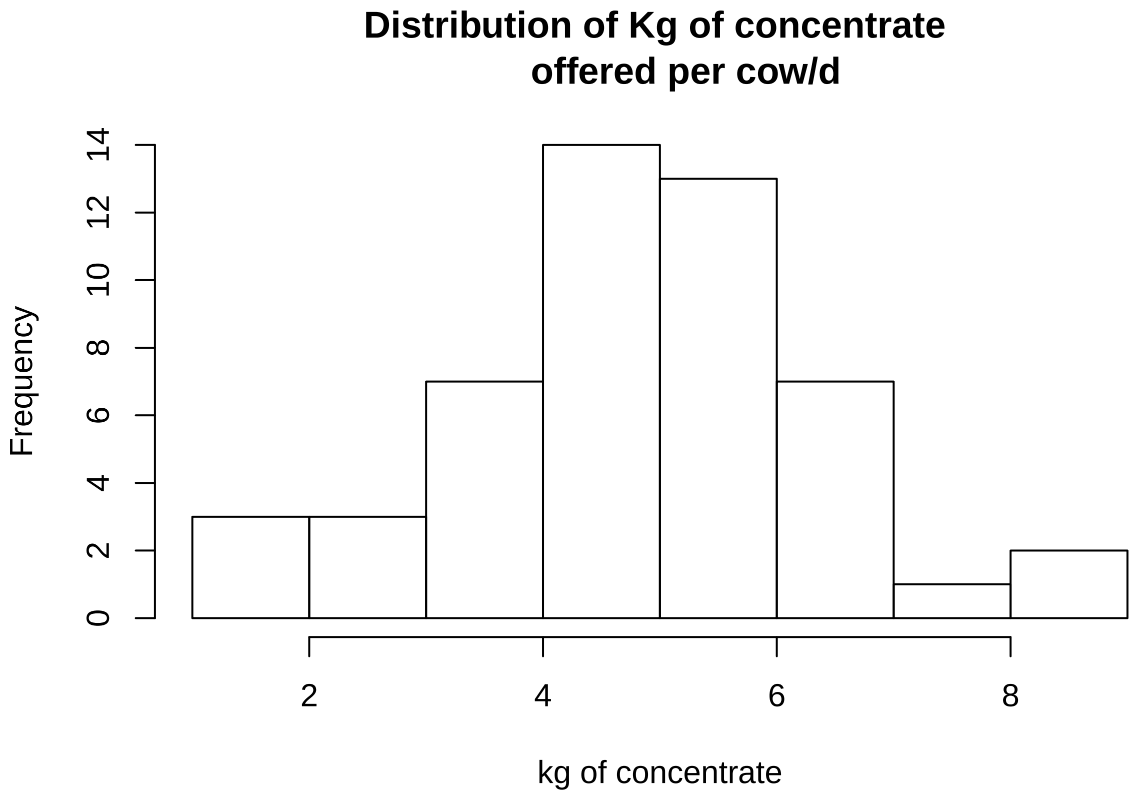


```
summary(DF$concentrate.kg.cow.d) #3 NAs
```

```
##    Min. 1st Qu.  Median    Mean 3rd Qu.    Max.    NA's
##   1.500   4.125   5.000   5.184   6.000   9.000       3
```

```
sd(DF$concentrate.kg.cow.d, na.rm = T)
```

```
## [1] 1.620374
```

Average amount of silage: Given per cow per day, as reported by farmer. *Farmers were very insecure on reporting this value. All farmers used corn silage.

```
hist(DF$silage.kg.cow, main = "Distribution of Kg of silage given per cow/d",
     xlab = "kg of silage")
```

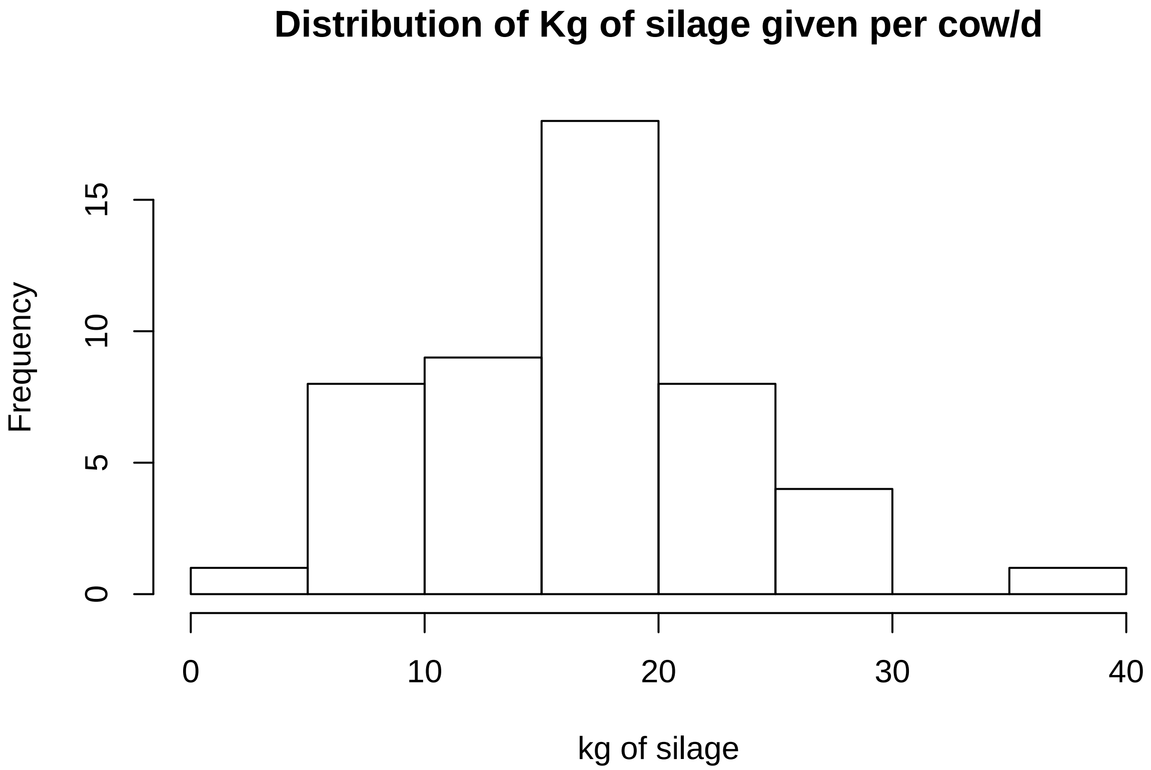


```
summary(DF$silage.kg.cow) #4 NAs
```

```
##    Min. 1st Qu.  Median    Mean 3rd Qu.    Max.    NA's
##    4.00   13.00   20.00   18.17   23.00   40.00       4
```

Area of silage per cow: Perhaps a more refined measurement of amount of silage given per cow per day, as no farmer reported buying silage from other sources and not giving silage to other animals (e.g., beef cows).

```
hist(DF$silage.area.cow, main = "Distribution of area of silage/cow",
     xlab = "Hectares of silage per cow/yr")
```

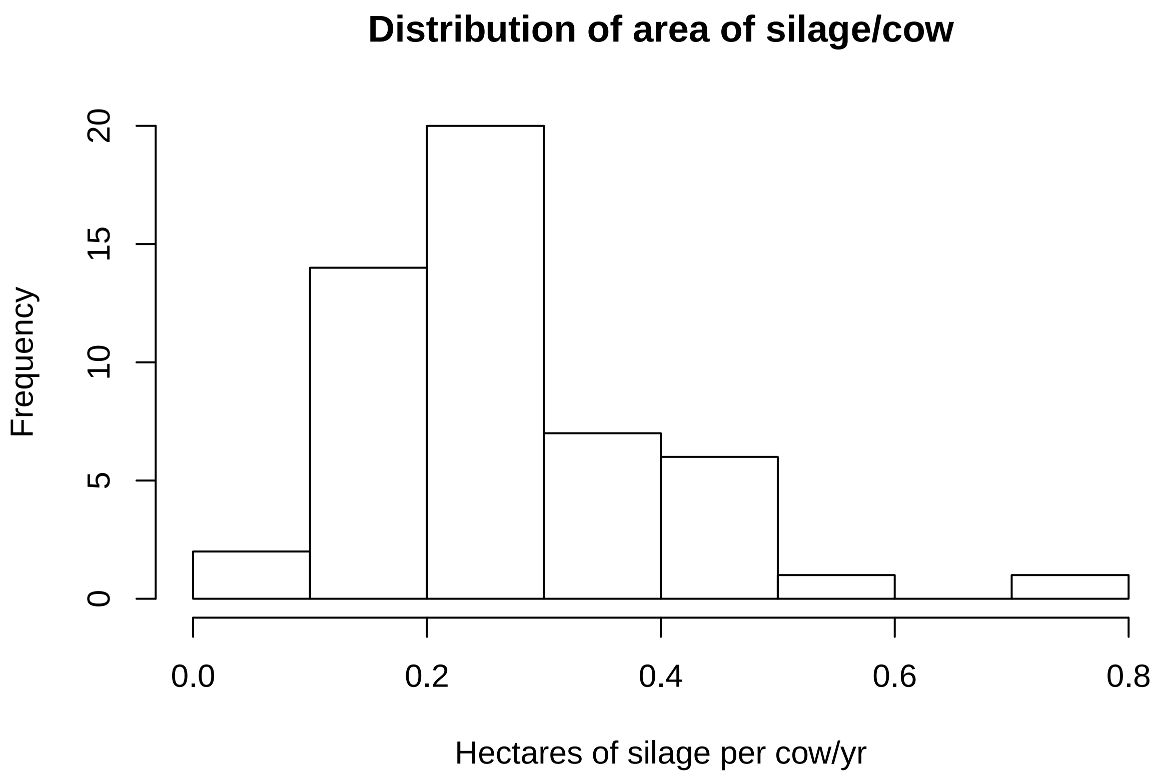


```
summary(DF$silage.area.cow) #2 NAs
```

```
##    Min. 1st Qu.  Median    Mean 3rd Qu.    Max.    NA's 
## 0.09091 0.20000 0.23636 0.27245 0.31099 0.76923       2
```

```
sd(DF$silage.area.cow, na.rm = T)
```

```
## [1] 0.1285124
```

Weather variables: Descriptive stats on weather variables

```
skimr::skim_with(numeric = list(hist = NULL)) #setting to drop hist on output
skimr::skim(select(weather, contains("temp")))
```

```
## Skim summary statistics
##  n obs: 53 
##  n variables: 3 
## 
## -- Variable type:numeric ----------------------------------------------------------------------------
##  variable missing complete  n  mean   sd    p0   p25   p50   p75  p100
##  avg_temp       1       52 53 20.21 3.88 11.41 17.74 19.57 23.84 25.96
##  max_temp       1       52 53 24.54 4.38 13.4  21.62 24.4  29    30.2 
##  min_temp       1       52 53 16.57 3.82  7.6  13.8  17.6  19.42 22.3
```

```
skimr::skim(select(weather, contains("hum")))
```

```
## Skim summary statistics
##  n obs: 53 
##  n variables: 3 
## 
## -- Variable type:numeric ----------------------------------------------------------------------------
##  variable missing complete  n  mean    sd    p0   p25   p50   p75   p100
```

```
##   avg_hum       1       52 53 77.69 11.28 57.21 67.46 77.88 84.96  97.58
##   max_hum       1       52 53 92.31  8.08 71    86    96.5  98    100
##   min_hum       1       52 53 59    15.45 34    50.5  56.5  66     94
```

```
skimr::skim(select(weather, contains("thi")))
```

```
## Skim summary statistics
##  n obs: 53
##  n variables: 6
##
## -- Variable type:numeric --------------------------------------------------------------------------------
##       variable missing complete  n  mean    sd    p0   p25   p50   p75
##        avg_THI       1       52 53 66.63  5.85 52.59 63.02 66.34 71.83
##    avg_THI_roll       1       52 53 67.3   4.63 55.34 63.7  68.24 71.31
##        max_THI       1       52 53 71.98  5.62 56.24 69.04 71.71 76.92
##        min_THI       1       52 53 61.51  6.52 45.82 56.9  63.62 66.3
##  sum_THI74_roll       1       52 53 12.98 13.38  0     0     8    26.5
##     THI74_hours       1       52 53  3.31  3.99  0     0     0     7
##   p100
##  74.9
##  72.93
##  79.34
##  70.51
##  34
##  11
```

Based on Linvill and Pardue, (1992), we have summed the number of hours of THI above 74 in the previous 4 day of the visit to assess the impact of heat stress on milk production. We also calculated 4 previous day avg THI. Both avg thi and hours THI74 will be tested, the variable that better explains the predictor will be used on the final model.

```
#Average THI 4 days previous visit day
summary(DF$avg_THI_roll)
```

```
##    Min. 1st Qu.  Median    Mean 3rd Qu.    Max.    NA's
##   55.34   63.70   68.24   67.30   71.31   72.93       1
```

```
hist(DF$avg_THI_roll)
```

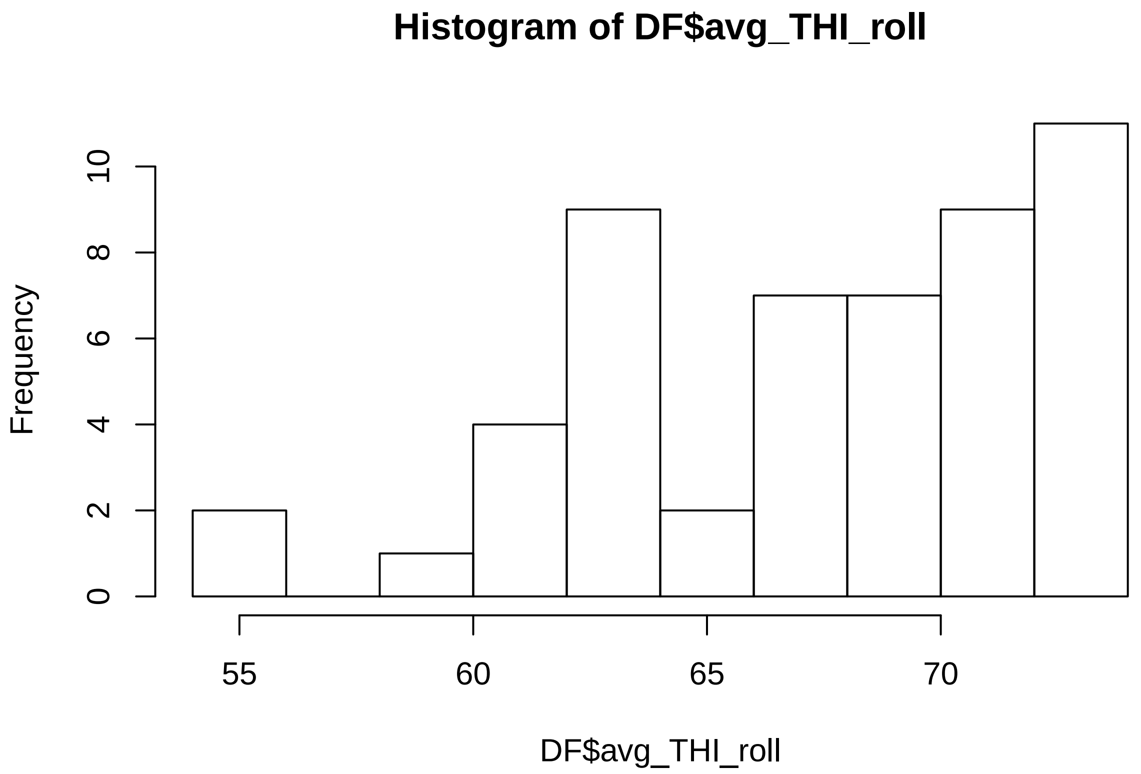


```r
#good variation

#Sum of hour above thi 74 in the 4 days previous visit day
summary(DF$sum_THI74_roll)
```

```
##    Min. 1st Qu.  Median    Mean 3rd Qu.    Max.    NA's 
##    0.00    0.00    8.00   12.98   26.50   34.00       1
```

```r
hist(DF$sum_THI74_roll)
```

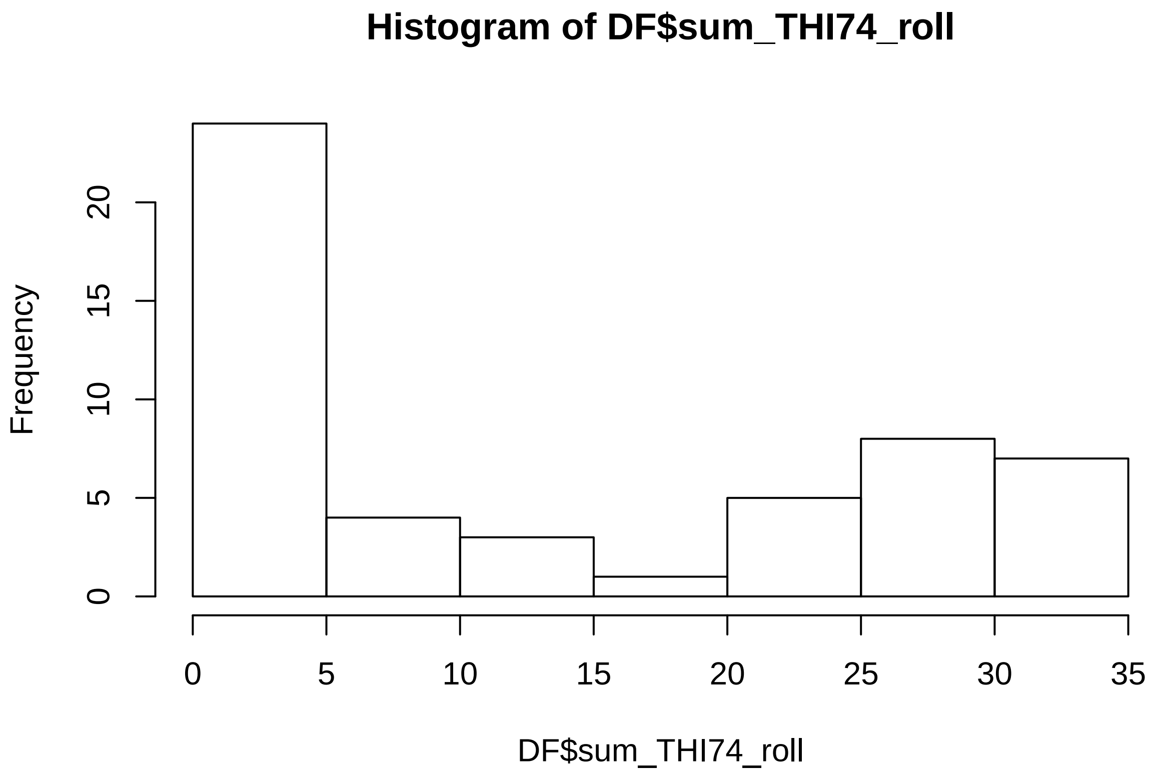


```
#good variation, not too high though.
```

Preciptation Precipitation may influence the amount of fresh forage consumed and thus effect either milk yield or water consumption.

```
#rain in mm in the day of the visit
summary(DF$precip)
```

```
##    Min. 1st Qu.  Median    Mean 3rd Qu.    Max.    NA's
##   0.000   0.000   0.200   2.354   1.800  18.600       1
```

```
hist(DF$precip)
```

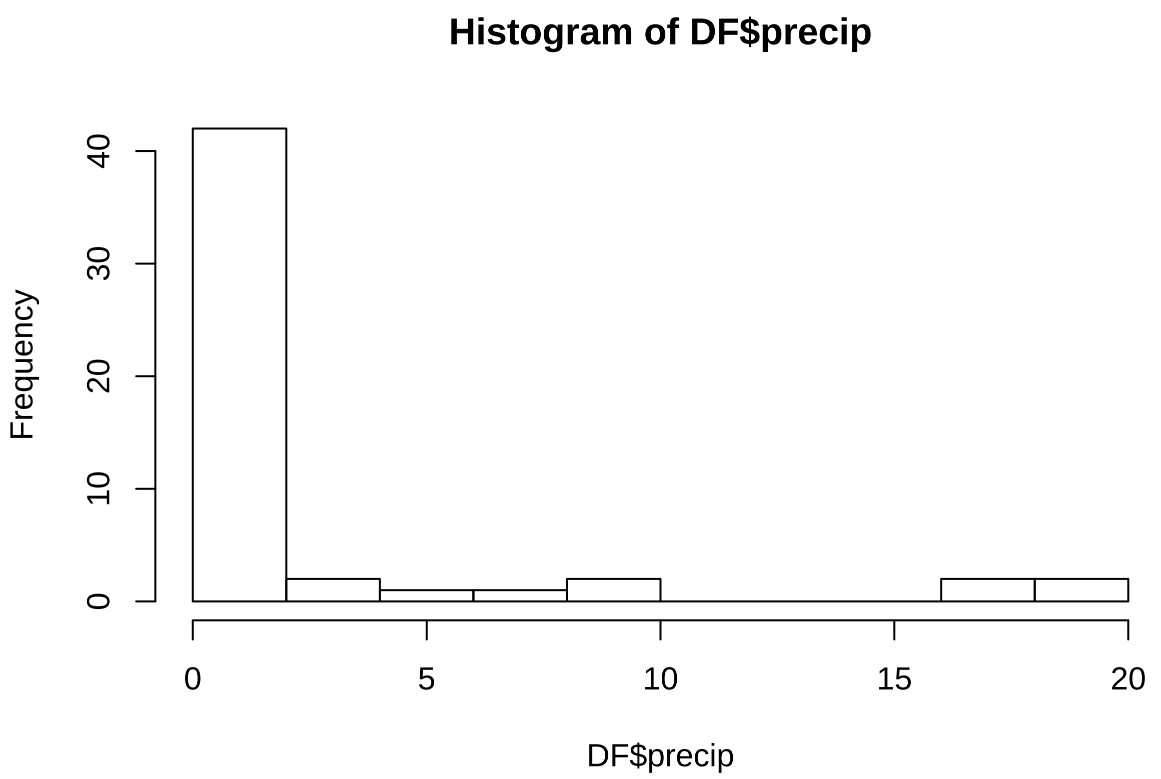


```
#cumulative rain 4 days before visit day
summary(DF$sum_precip_4d)
```

```
##    Min. 1st Qu.  Median    Mean 3rd Qu.    Max.    NA's 
##    0.00    0.35    5.00   23.63   29.30  167.60       1
```

```
hist(DF$sum_precip_4d)
```

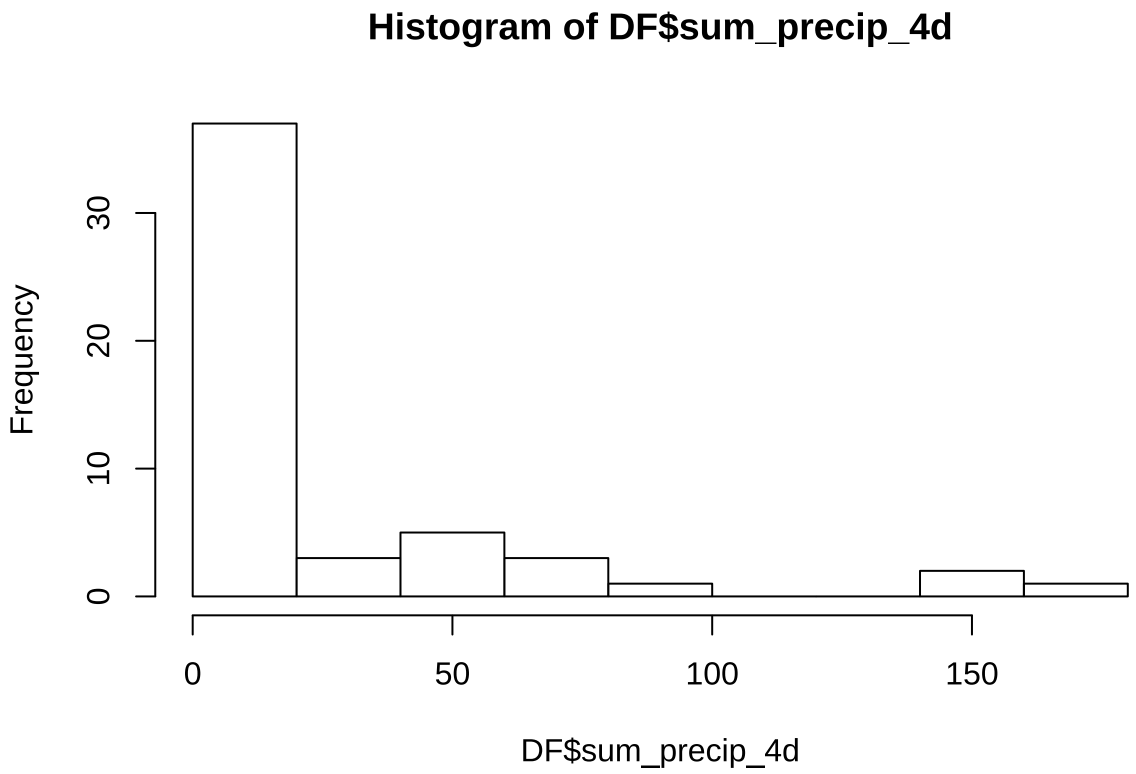

2.1.3.2 Categorical variables

Breed: As being Holstein only (>75% Holstein), or non-Holstein herd. Median for the varibale percetage of holsteins in the herd = 0.75, thus it makes sense to cut at >75.

```
DF$mainbreed <- ifelse(DF$holpercent > 0.75, "holstein","non-holstein")
table(DF$mainbreed)
```

```
##
##     holstein non-holstein
##           26           27
```

Number of paddocks per day: Categorical variable, because we only have one farms that provided 3 fresh paddocks/d for the herd, we need to create a two-level categorical variable as 1 paddock/d and ??? 2paddocks/day.

```
table(DF$hours.paddock)# 8 = 3paddocks/day; 12 = 2paddocks/day; 24 = 1paddock/day
```

```
##
##  8 12 24
##  1 43  8
```

```
#create binary variable 2 vs 1 paddock per day
DF$hours.paddock[DF$hours.paddock==8] <- "Two"
DF$hours.paddock[DF$hours.paddock==12] <- "Two"
DF$hours.paddock[DF$hours.paddock==24] <- "One"

#renaming to consider changes
colnames(DF)[colnames(DF)=="hours.paddock"] <- "paddocks.d"

table(DF$paddocks.d) #too few farms use only 1 paddock per day
```

```
##
## One Two
##   8  44
```

```r
table(DF$paddocks.d, DF$water.access) #let's test
```

```
##
##       free limited
##   One    3       5
##   Two   24      20
```

```r
fisher.test(DF$water.access, DF$paddocks.d) #No evidence that it's unbalanced
```

```
##
##  Fisher's Exact Test for Count Data
##
## data:  DF$water.access and DF$paddocks.d
## p-value = 0.4583
## alternative hypothesis: true odds ratio is not equal to 1
## 95 percent confidence interval:
##  0.06991945 2.97990568
## sample estimates:
## odds ratio
##  0.5066987
```

Due to this variable being balanced across water type groups it will not be further considered for modelling.

Number of feedings per day: Number of times per day cows were given silage

```r
table(DF$silage.freq.d)
```

```
##
##  1  2  3
## 12 30  8
```

There is a good variation across this varibles. We will explore it further in the section below.

Pasture maturity: Before optimal cutting point (no presence of flowers and/or no senescent basal leaves), optimal cutting point (no presence of flowers and 1 to 3 leaves senescent) and after optimal cutting point (presence of flowers or >3 senescent basal leaves)

```r
table(DF$pasture.maturity)
```

```
##
##  optimum post-opt  pre-opt
##       18       18        9
```

There is a good variation across this varible. We will explore it further in the section below.

Preciptation Precipitation data will be categorized into any rain during the 4 days before vist or no rain.

```r
table(DF$precip_cat)
```

```
##
##  dry rain
##    9   43
```

It rained pretty much in all farms during the study.

2.1.4 Unconditional modelling

This approach will be used to screen for possible cow-level or environmental-level confounding variables that should be included in the multivariate model to measure the associations of management practices with milk yield. Variables will be considered for multivariable model if $p<0.2$.

Outcome variable is normally distributed, thus no transformation is needed before hand. Linear regression models will be used to measure the association between variables.

Model assumptions will be assessed graphically.

```
#drop any farm that has no data on water access
DF2 <- DF[complete.cases(DF$water.access), ]
```

Breed - continuous

```
breedcont <- lm(avg.cow.d ~ holpercent, data = DF2)
summary(breedcont)
```

```
##
## Call:
## lm(formula = avg.cow.d ~ holpercent, data = DF2)
##
## Residuals:
##     Min      1Q  Median      3Q     Max
## -9.3439 -2.3071 -0.0157  1.9387  9.4076
##
## Coefficients:
##             Estimate Std. Error t value Pr(>|t|)
## (Intercept)   13.168      1.209  10.896 1.42e-14 ***
## holpercent     6.440      1.584   4.066 0.000177 ***
## ---
## Signif. codes:  0 '***' 0.001 '**' 0.01 '*' 0.05 '.' 0.1 ' ' 1
##
## Residual standard error: 3.502 on 48 degrees of freedom
##   (2 observations deleted due to missingness)
## Multiple R-squared:  0.2562, Adjusted R-squared:  0.2407
## F-statistic: 16.53 on 1 and 48 DF,  p-value: 0.0001768
```

```
scatterplot(avg.cow.d ~ holpercent, data = DF2)
```

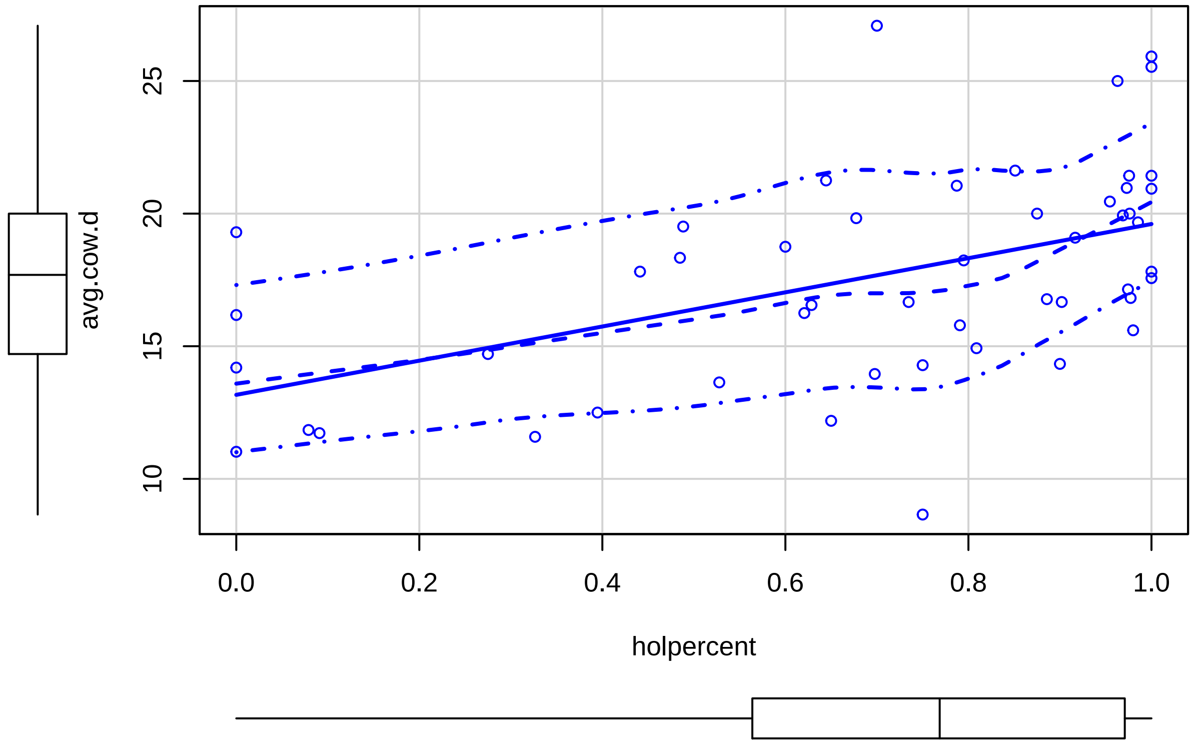


```
residualPlot(breedcont, quadratic = FALSE)
```

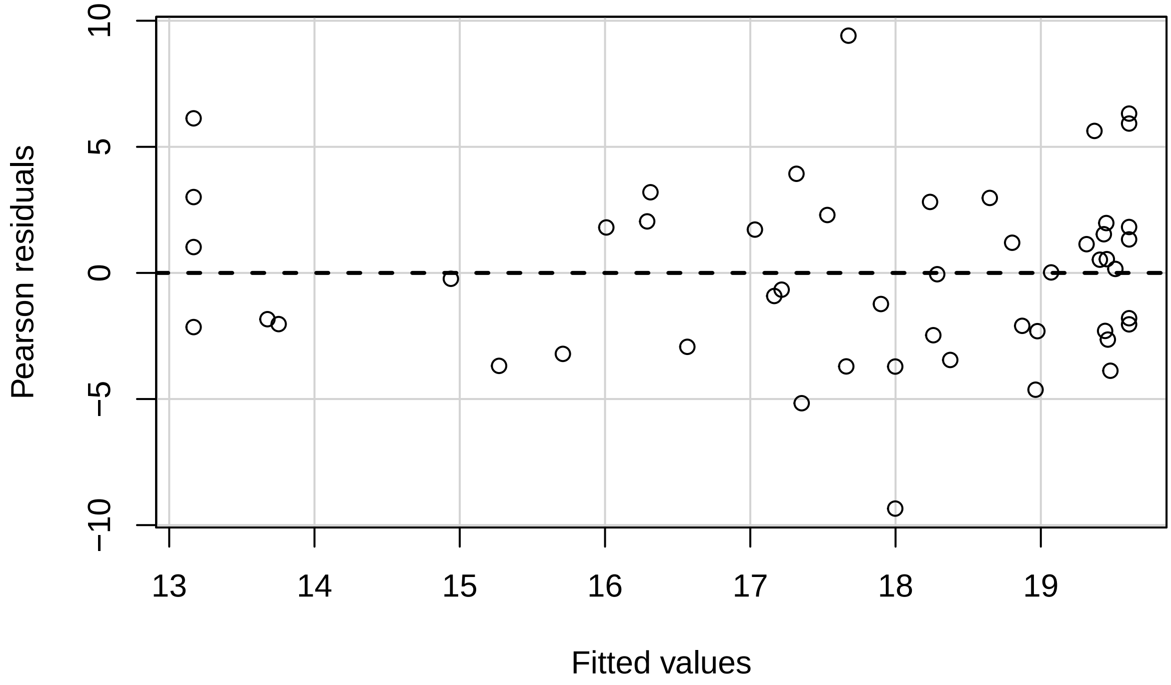

As expected, the percentage of Holstein cows in the herd are positively associated with herd milk production

Breed - categorical

```
breedcat <- lm(avg.cow.d ~ mainbreed, data = DF2)
summary(breedcat)
```

```
##
```

```
## Call:
## lm(formula = avg.cow.d ~ mainbreed, data = DF2)
##
## Residuals:
##     Min      1Q  Median      3Q     Max
## -7.0878 -2.6267  0.3475  2.0167 11.3416
##
## Coefficients:
##                         Estimate Std. Error t value Pr(>|t|)
## (Intercept)              19.4119     0.7066  27.472  < 2e-16 ***
## mainbreednon-holstein  -3.6702     1.0199  -3.599 0.000755 ***
## ---
## Signif. codes:  0 '***' 0.001 '**' 0.01 '*' 0.05 '.' 0.1 ' ' 1
##
## Residual standard error: 3.603 on 48 degrees of freedom
##   (2 observations deleted due to missingness)
## Multiple R-squared:  0.2125, Adjusted R-squared:  0.1961
## F-statistic: 12.95 on 1 and 48 DF,  p-value: 0.0007553
```

```
qqPlot(breedcat)
```

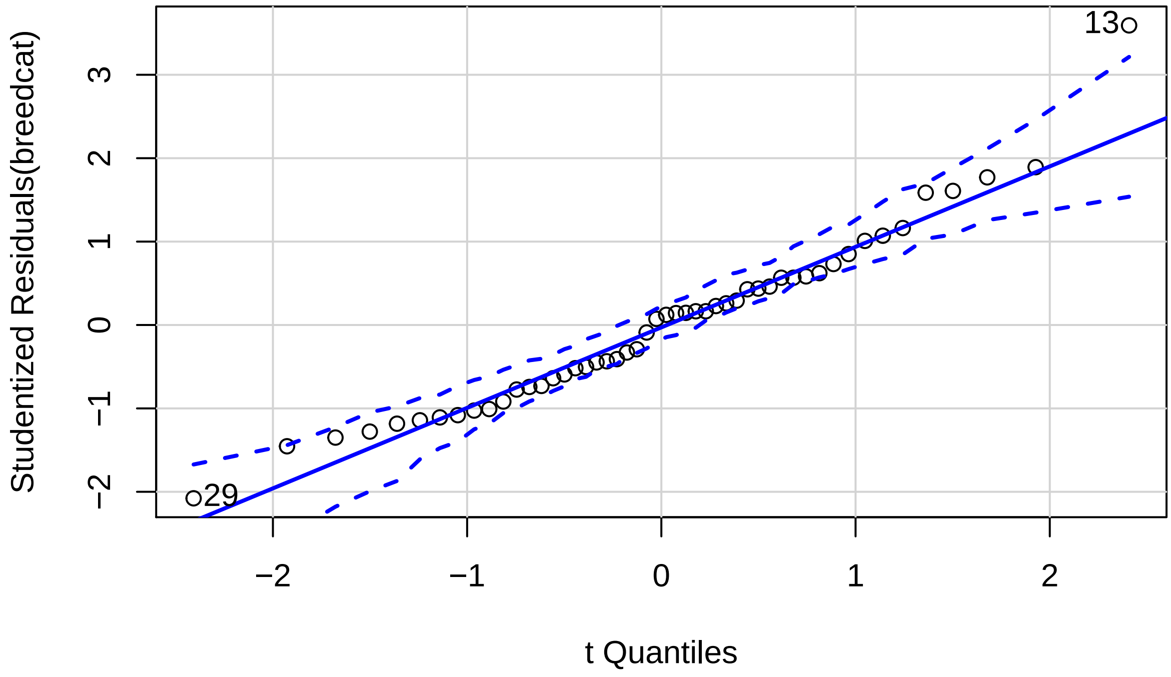


```
## [1] 13 29
```

```
residualPlot(breedcat, quadratic = FALSE)
```

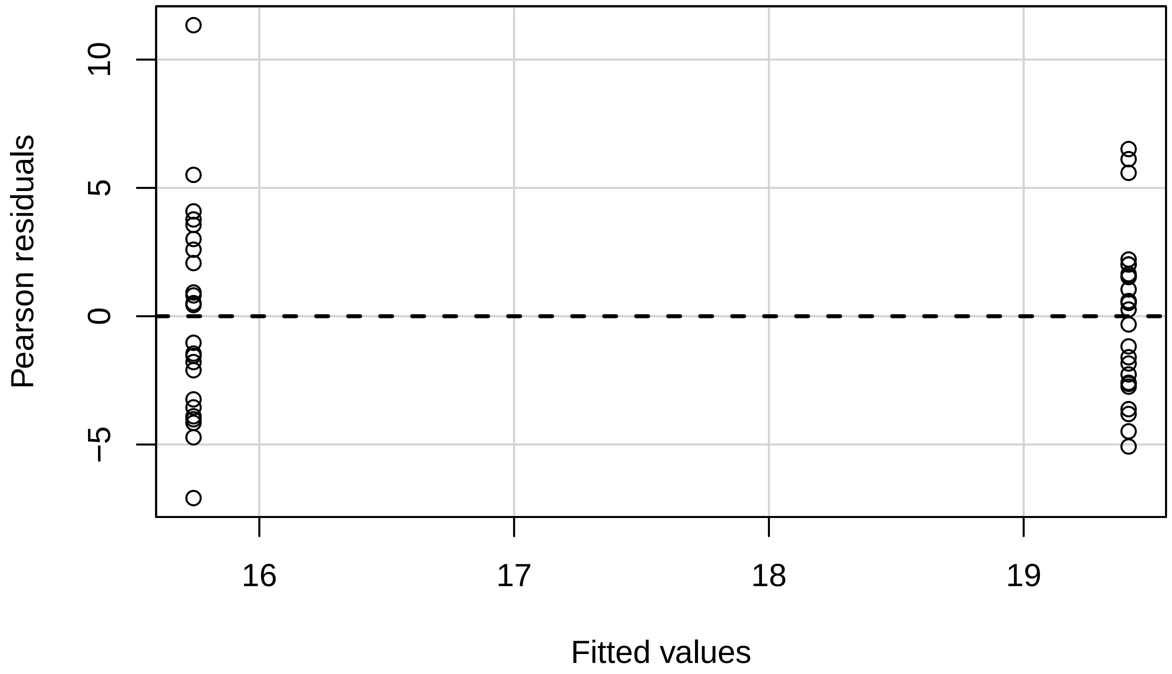

Categorizing herds by breed also yields a significant effect of breed on herd milk yield.

Below is an ANOVA to compare which model fits best.

```
anova(breedcat, breedcont)
```

```
## Analysis of Variance Table
##
## Model 1: avg.cow.d ~ mainbreed
## Model 2: avg.cow.d ~ holpercent
##   Res.Df    RSS Df Sum of Sq F Pr(>F)
## 1     48 623.14
## 2     48 588.54  0    34.595
```

Models do not differ in their fit (very similar RSS), so I decided to keep the categorical variable. The focus of this paper is not to explore the effect of breeds on milk yield but to include in the model to control for the confounding effect on milk yield and to adjust the estimates for the water access variable.

Amount of concentrate

```
conc <- lm(avg.cow.d ~ concentrate.kg.cow.d, data = DF2)
summary(conc)
```

```
##
## Call:
## lm(formula = avg.cow.d ~ concentrate.kg.cow.d, data = DF2)
##
## Residuals:
##     Min      1Q  Median      3Q     Max
## -5.4632 -1.8626 -0.1068  2.1265  7.8282
##
## Coefficients:
##                      Estimate Std. Error t value Pr(>|t|)
## (Intercept)            9.5419     1.3996   6.818 1.54e-08 ***
## concentrate.kg.cow.d   1.5260     0.2575   5.925 3.49e-07 ***
```

```
## ---
## Signif. codes:  0 '***' 0.001 '**' 0.01 '*' 0.05 '.' 0.1 ' ' 1
##
## Residual standard error: 2.921 on 47 degrees of freedom
##   (3 observations deleted due to missingness)
## Multiple R-squared:  0.4276, Adjusted R-squared:  0.4154
## F-statistic: 35.11 on 1 and 47 DF,  p-value: 3.492e-07
```

```
qqPlot(conc)
```

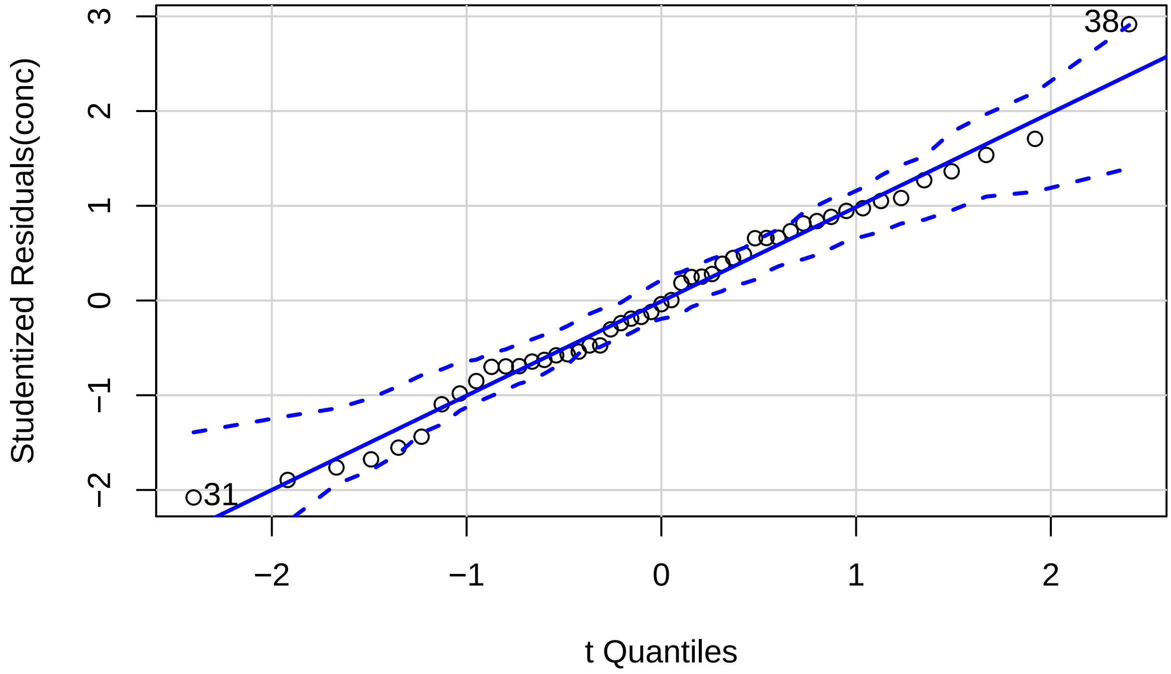


```
## [1] 31 38
```

```
residualPlot(conc, quadratic = FALSE)
```

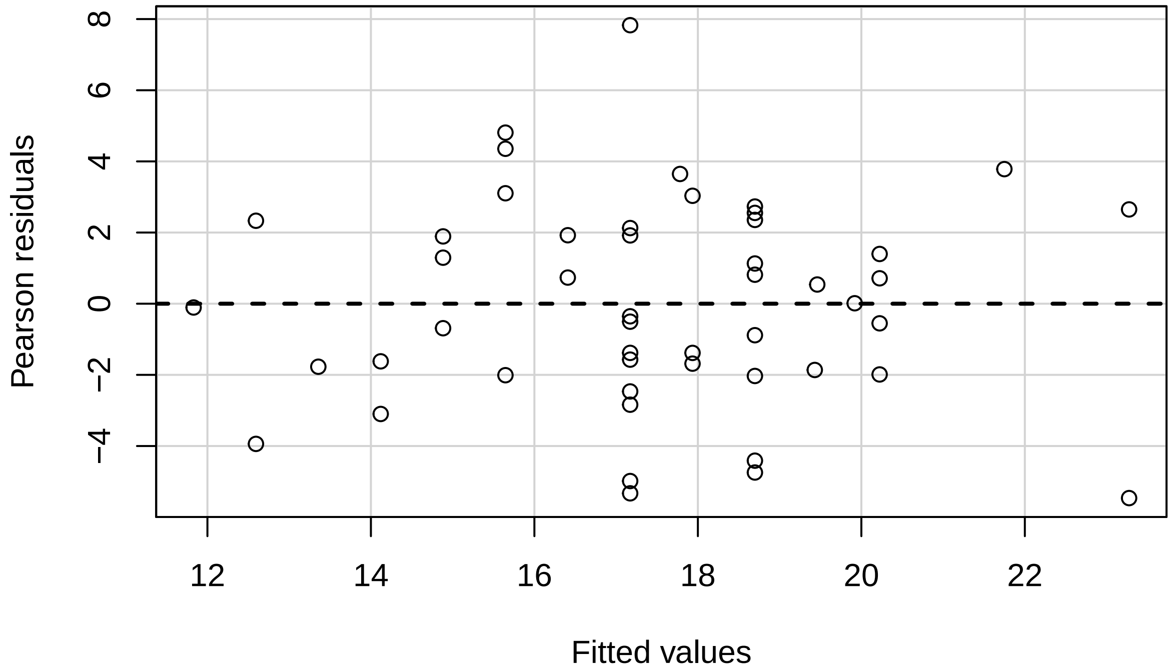


```r
scatterplot(DF$avg.cow.d ~ DF$concentrate.kg.cow.d)
```

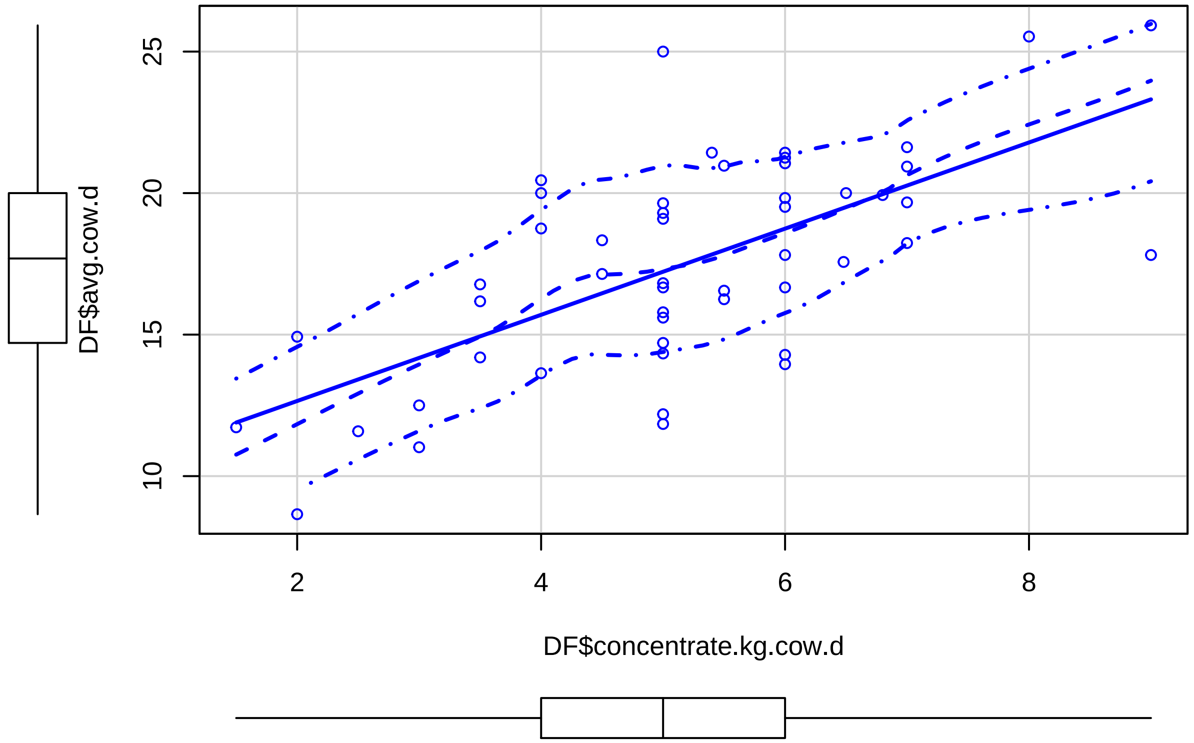

Model fits and variable is highly associated with the outcome, thus this variable must be included in the final model.

Amount of silage

```r
sil <- lm(avg.cow.d ~ silage.kg.cow, data = DF2)
summary(sil)
```

```
##
## Call:
## lm(formula = avg.cow.d ~ silage.kg.cow, data = DF2)
##
## Residuals:
##     Min      1Q  Median      3Q     Max
## -6.5907 -2.8721 -0.1565  2.2262  9.2922
##
## Coefficients:
##               Estimate Std. Error t value Pr(>|t|)
## (Intercept)   17.51415    1.50324  11.651 2.54e-15 ***
## silage.kg.cow  0.01385    0.07644   0.181    0.857
## ---
## Signif. codes:  0 '***' 0.001 '**' 0.01 '*' 0.05 '.' 0.1 ' ' 1
##
## Residual standard error: 3.894 on 46 degrees of freedom
##   (4 observations deleted due to missingness)
## Multiple R-squared:  0.0007129,  Adjusted R-squared:  -0.02101
## F-statistic: 0.03282 on 1 and 46 DF,  p-value: 0.857
```

```
qqPlot(sil)
```

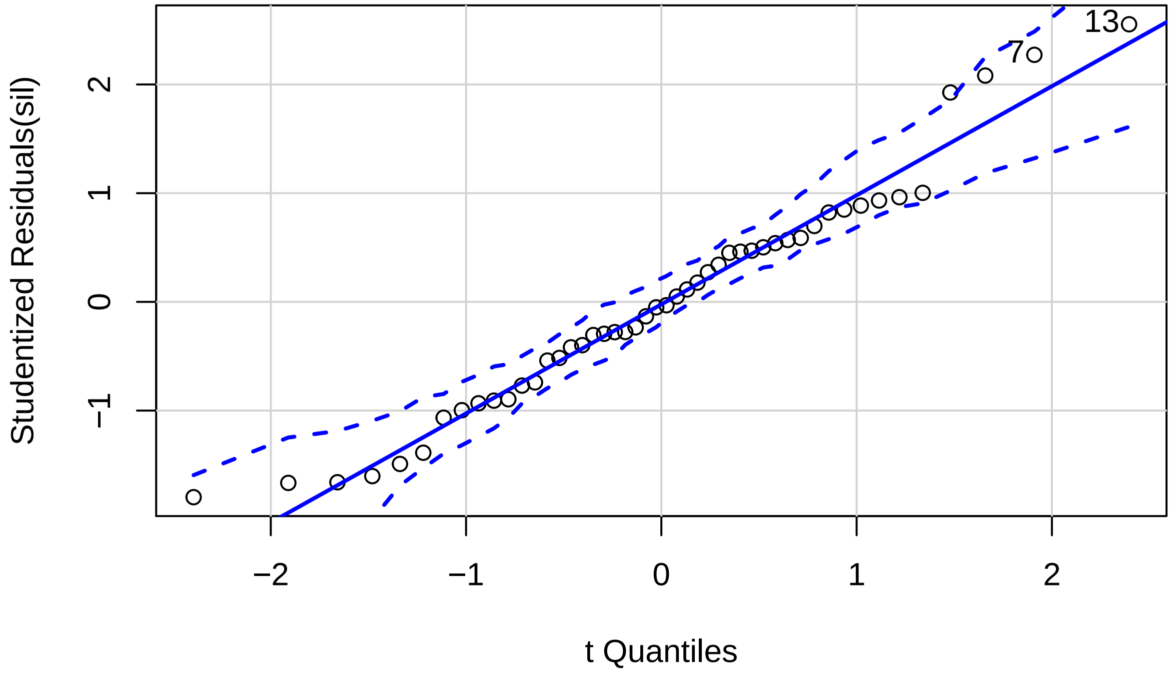


```
## [1]  7 13
```

```
residualPlot(sil, quadratic = FALSE)
```

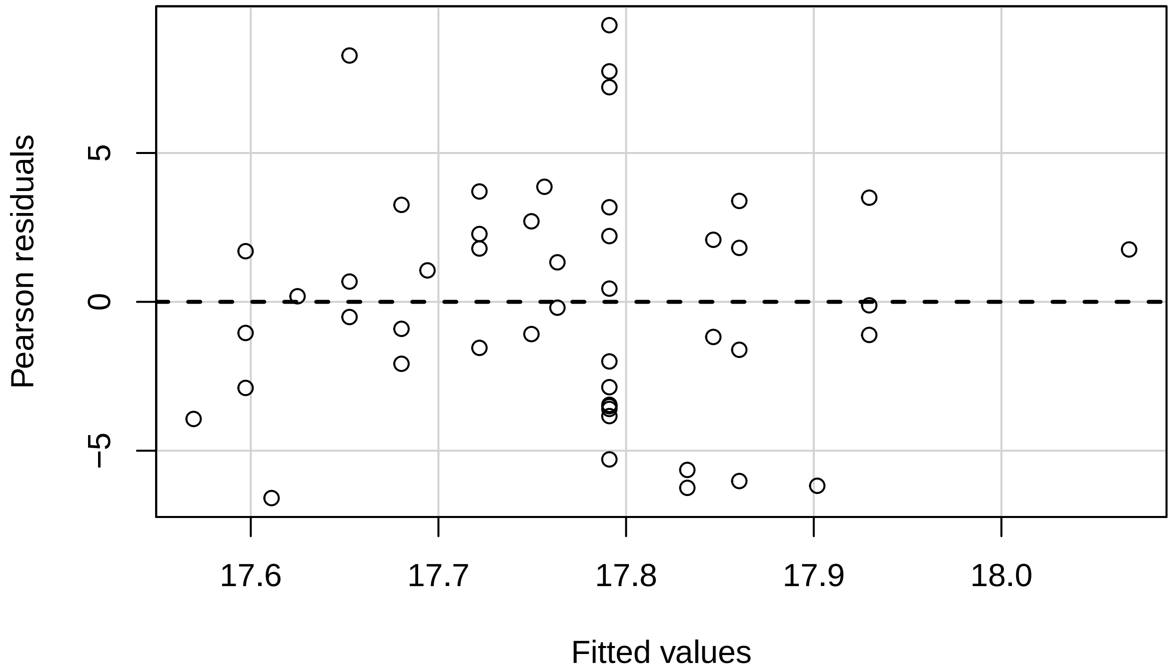


```
scatterplot(avg.cow.d ~ silage.kg.cow, data = DF2)
```

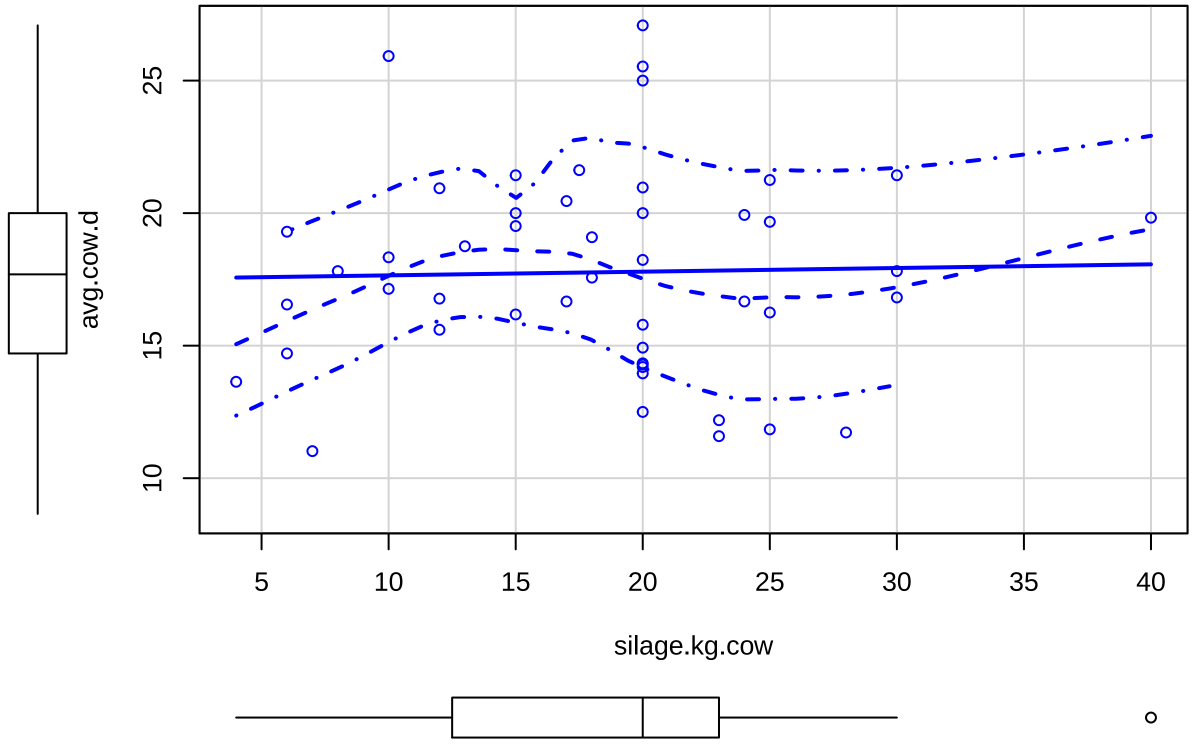

Model fits but variable is not associated with the outcome. Due to the fact that we haven't measured it, it's possible that the amount estimated by the farmer isn't representative of the actual amount offered to the cows. Below we will use other variables related to amount of silage offered to be used as a proxy for diet's silage content.

Number of silage feeding per day

Feeding silage more frequently per day may indicate that cows may consume more silage, thus increasing

milk yield.

```
f.sil <- lm(avg.cow.d ~ silage.freq.d, data = DF2)
summary(f.sil)
```

```
##
## Call:
## lm(formula = avg.cow.d ~ silage.freq.d, data = DF2)
##
## Residuals:
##     Min      1Q  Median      3Q     Max
## -7.3158 -2.8062  0.2804  2.0296  9.5623
##
## Coefficients:
##                  Estimate Std. Error t value Pr(>|t|)
## (Intercept)        15.970      1.155  13.828   <2e-16 ***
## silage.freq.d2      2.001      1.373   1.457    0.152
## silage.freq.d3      2.626      1.826   1.438    0.157
## ---
## Signif. codes:  0 '***' 0.001 '**' 0.01 '*' 0.05 '.' 0.1 ' ' 1
##
## Residual standard error: 4.001 on 46 degrees of freedom
##   (3 observations deleted due to missingness)
## Multiple R-squared:  0.05614,    Adjusted R-squared:  0.0151
## F-statistic: 1.368 on 2 and 46 DF,  p-value: 0.2648
```

```
qqPlot(f.sil)
```

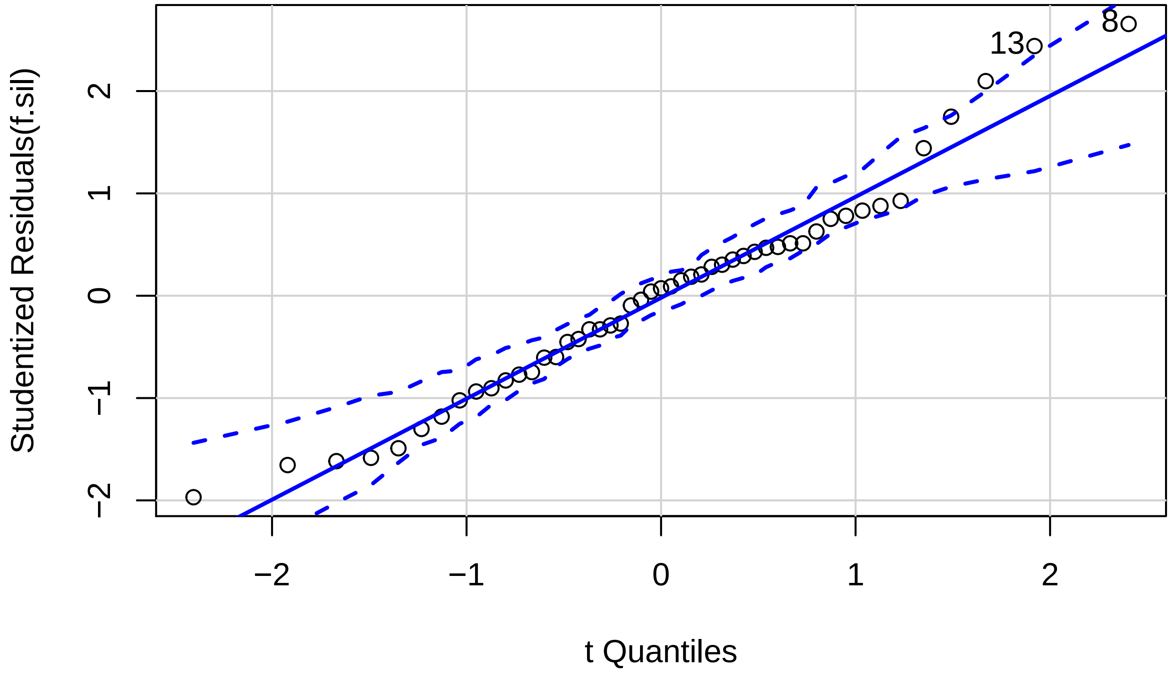


```
## [1]  8 13
```

```
residualPlot(f.sil, quadratic = FALSE)
```

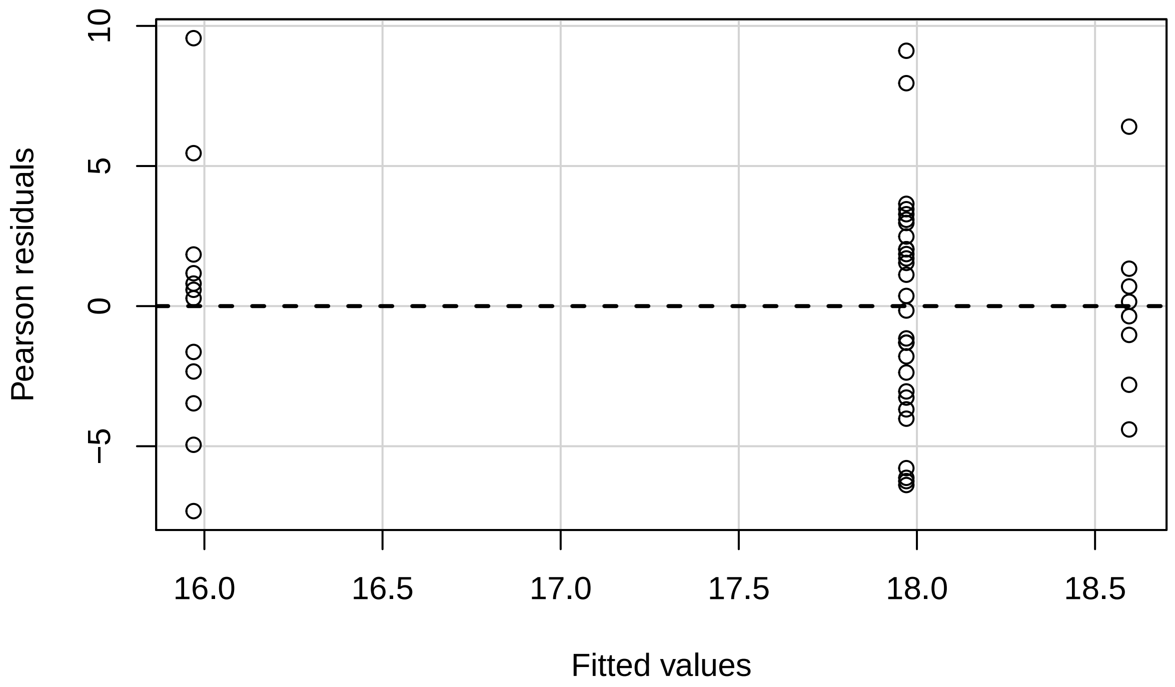

Model fits; variable slightly associated (p=.12) with the outcome. The direction of the association also fits with our hypothesis that more feeding = more milk. This may be a better proxy for amount of silage.

Area of silage per cow

```
a.sil <- lm(avg.cow.d ~ silage.area.cow, data = DF2)
summary(a.sil)
```

```
##
## Call:
## lm(formula = avg.cow.d ~ silage.area.cow, data = DF2)
##
## Residuals:
##     Min      1Q  Median      3Q     Max
## -9.3263 -2.5107 -0.1343  2.4593  9.6227
##
## Coefficients:
##                 Estimate Std. Error t value Pr(>|t|)
## (Intercept)       15.441      1.305   11.83 7.77e-16 ***
## silage.area.cow    8.080      4.320    1.87   0.0675 .
## ---
## Signif. codes:  0 '***' 0.001 '**' 0.01 '*' 0.05 '.' 0.1 ' ' 1
##
## Residual standard error: 3.92 on 48 degrees of freedom
##   (2 observations deleted due to missingness)
## Multiple R-squared:  0.06793,    Adjusted R-squared:  0.04851
## F-statistic: 3.498 on 1 and 48 DF,  p-value: 0.06754
```

```
qqPlot(a.sil)
```

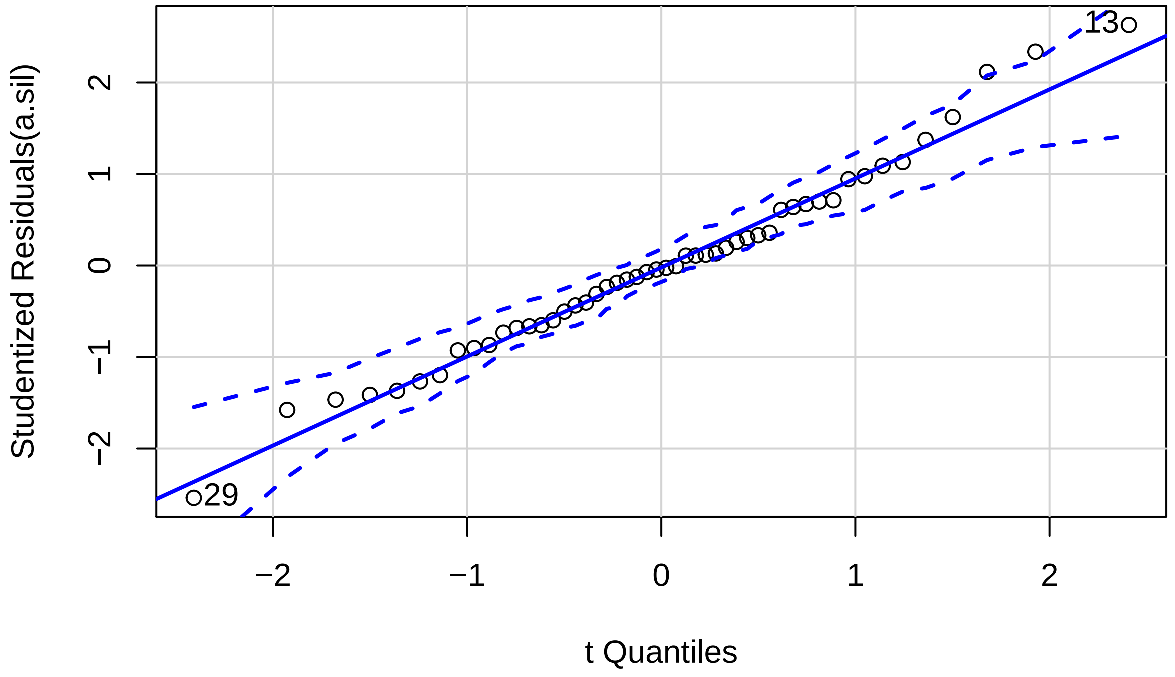


```
## [1] 13 29
```

```r
residualPlot(a.sil, quadratic = FALSE)
```

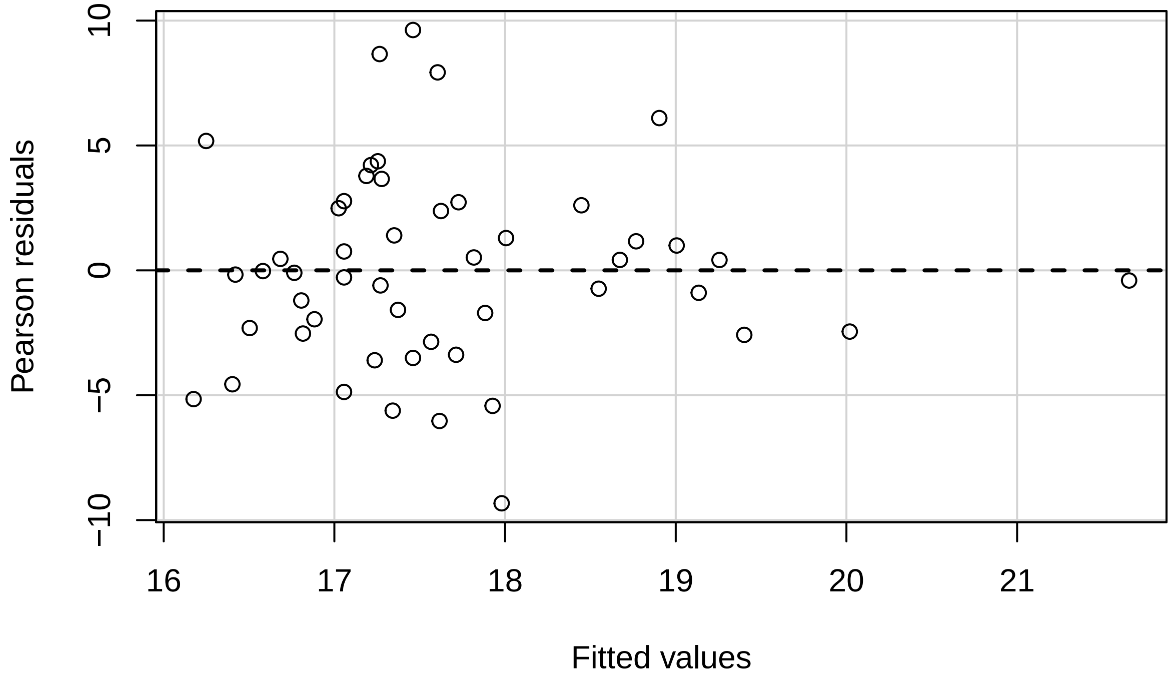


```r
scatterplot(avg.cow.d ~ silage.area.cow, data = DF2)
```

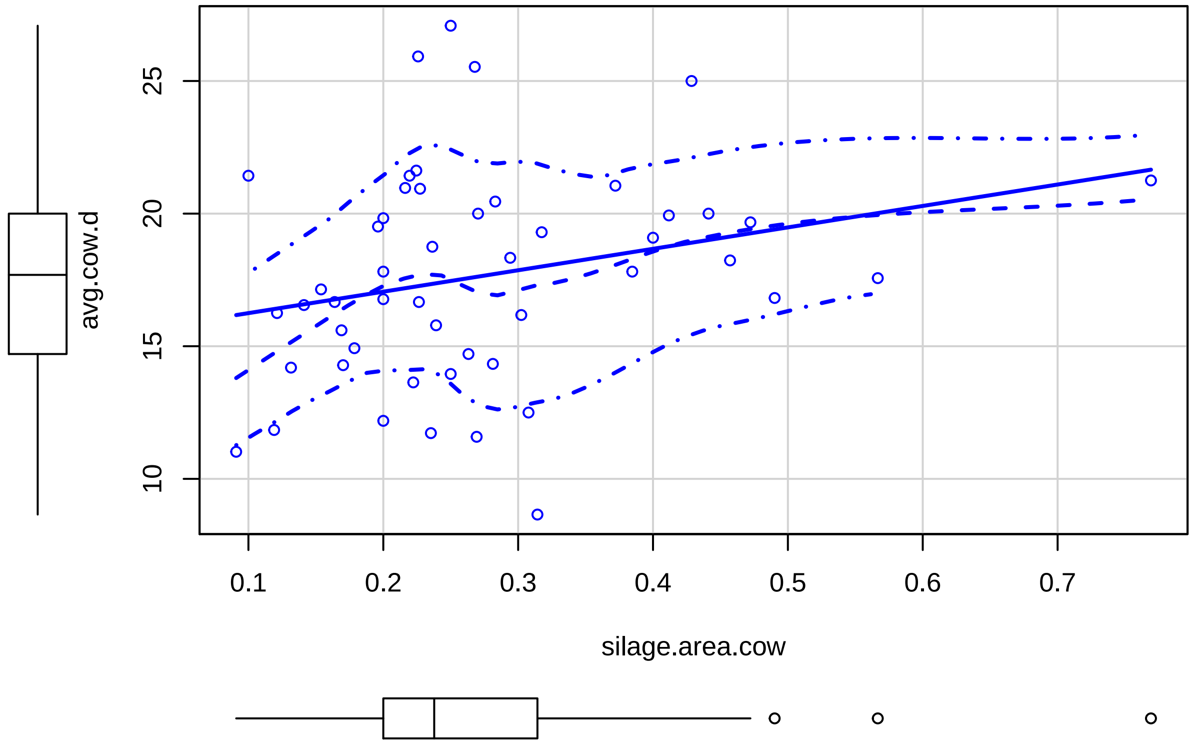

Model somewhat fits and variable is slightly associated with the outcome. Now we happen to have two variables regarding silage that might be a useful proxy for the amount of silage per day, are they correlated?

```
summary(lm(silage.area.cow ~ silage.freq.d, data = DF2))
```

```
##
## Call:
## lm(formula = silage.area.cow ~ silage.freq.d, data = DF2)
##
## Residuals:
##      Min       1Q   Median       3Q      Max
## -0.21701 -0.08096 -0.01780  0.07125  0.48827
##
## Coefficients:
##                Estimate Std. Error t value Pr(>|t|)
## (Intercept)     0.21000    0.03624   5.795 5.86e-07 ***
## silage.freq.d2  0.07096    0.04309   1.647   0.1064
## silage.freq.d3  0.13859    0.05729   2.419   0.0196 *
## ---
## Signif. codes:  0 '***' 0.001 '**' 0.01 '*' 0.05 '.' 0.1 ' ' 1
##
## Residual standard error: 0.1255 on 46 degrees of freedom
##   (3 observations deleted due to missingness)
## Multiple R-squared:  0.1159, Adjusted R-squared:  0.07749
## F-statistic: 3.016 on 2 and 46 DF,  p-value: 0.05878
```

r-sq = 8% so they are not strongly correlated. As freq of silage feeding is not normally dist I can’t do a correlation test, that’s why I’ve used lm.

Pasture maturity

Rational: optimum cutting point should lead to greater milk yield, however pre-opt pasture have higher

moisture content, thus if you have high moisture the effect of water trough location should be minimal under pre-opt pasture conditions.

```
pastu.m <- lm(avg.cow.d ~ pasture.optimum, data = DF2)
summary(pastu.m)
```

```
##
## Call:
## lm(formula = avg.cow.d ~ pasture.optimum, data = DF2)
##
## Residuals:
##     Min      1Q  Median      3Q     Max
## -8.4910 -2.8152 -0.1951  2.6525  9.9385
##
## Coefficients:
##                 Estimate Std. Error t value Pr(>|t|)
## (Intercept)      17.1449     0.8313   20.62   <2e-16 ***
## pasture.optimum   1.2855     1.2849    1.00    0.323
## ---
## Signif. codes:  0 '***' 0.001 '**' 0.01 '*' 0.05 '.' 0.1 ' ' 1
##
## Residual standard error: 4.157 on 41 degrees of freedom
##   (9 observations deleted due to missingness)
## Multiple R-squared:  0.02383,    Adjusted R-squared:  2.116e-05
## F-statistic: 1.001 on 1 and 41 DF,  p-value: 0.323
```

```
qqPlot(pastu.m)
```

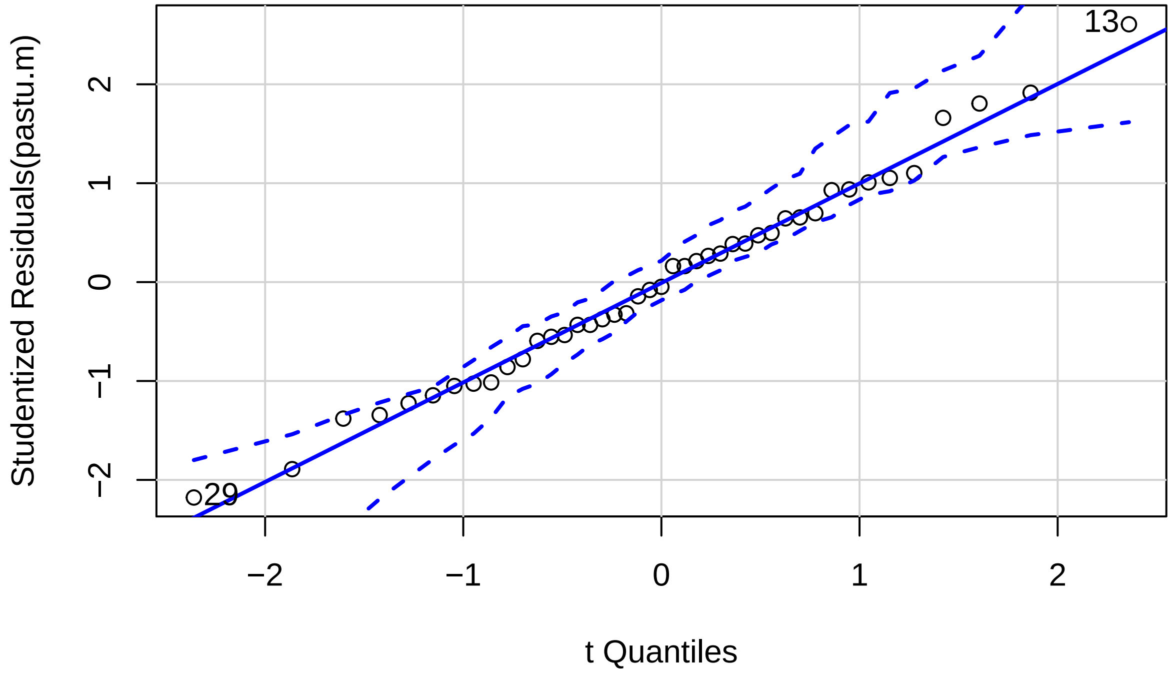


```
## [1] 13 29
```

```
residualPlot(pastu.m, quadratic = FALSE)
```

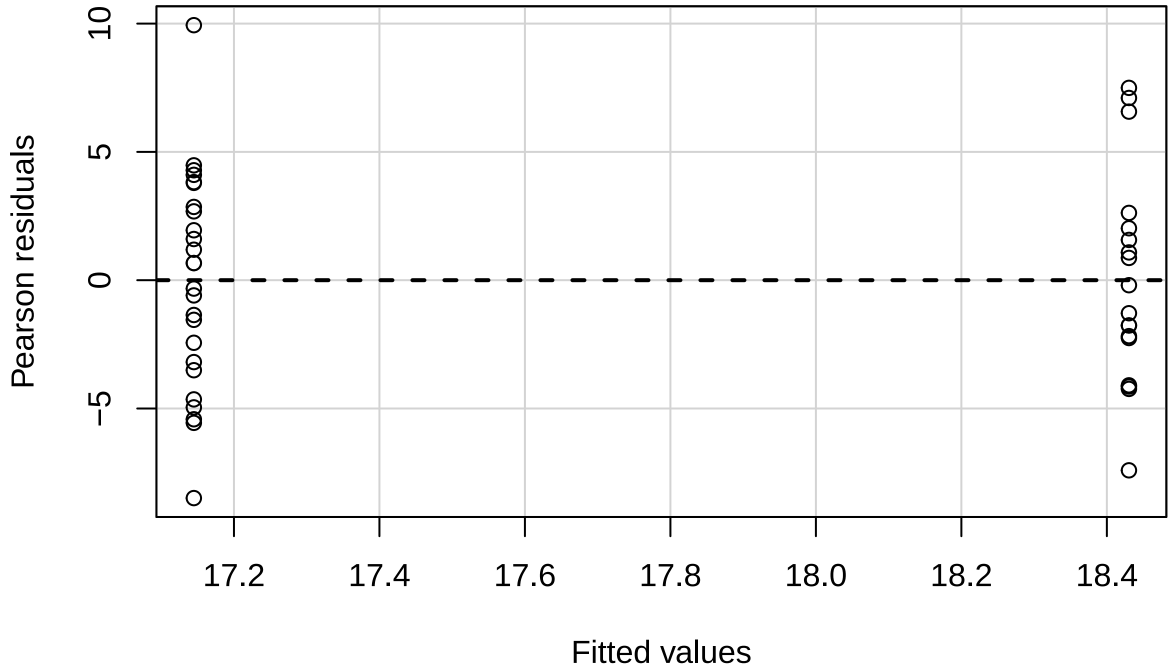


```
#no effect of pasture maturity on milk yield, note that here I am testing the
#hypothesis that only optimum cutting point yields greater milk production,
#that is why pre-opt and post-opt are combined as the referrent parameter
#(intercept)

table(DF2$pasture.pre.opt, DF2$water.access) #number of obs per cell
```

```
##
##     free limited
##   0   20      16
##   1    4       5
```

```
# seems that we have enough data to model the interactions

pastu.m2 <- lm(avg.cow.d ~ pasture.pre.opt * water.access, data = DF2)
summary(pastu.m2)
```

```
##
## Call:
## lm(formula = avg.cow.d ~ pasture.pre.opt * water.access, data = DF2)
##
## Residuals:
##     Min      1Q  Median      3Q     Max
## -6.8868 -3.0487 -0.1388  2.4485  9.5151
##
## Coefficients:
##                                       Estimate Std. Error t value Pr(>|t|)
## (Intercept)                            18.4722     0.9382  19.689   <2e-16
## pasture.pre.opt                        -3.0154     2.2981  -1.312    0.197
## water.accesslimited                    -0.9039     1.4331  -0.631    0.532
## pasture.pre.opt:water.accesslimited     1.8404     3.2948   0.559    0.580
##
## (Intercept)                           ***
```

```
## pasture.pre.opt
## water.accesslimited
## pasture.pre.opt:water.accesslimited
## ---
## Signif. codes:  0 '***' 0.001 '**' 0.01 '*' 0.05 '.' 0.1 ' ' 1
##
## Residual standard error: 4.196 on 39 degrees of freedom
##   (9 observations deleted due to missingness)
## Multiple R-squared:  0.05392,    Adjusted R-squared:  -0.01886
## F-statistic: 0.7409 on 3 and 39 DF,  p-value: 0.5341
```

```r
qqPlot(pastu.m2)
```

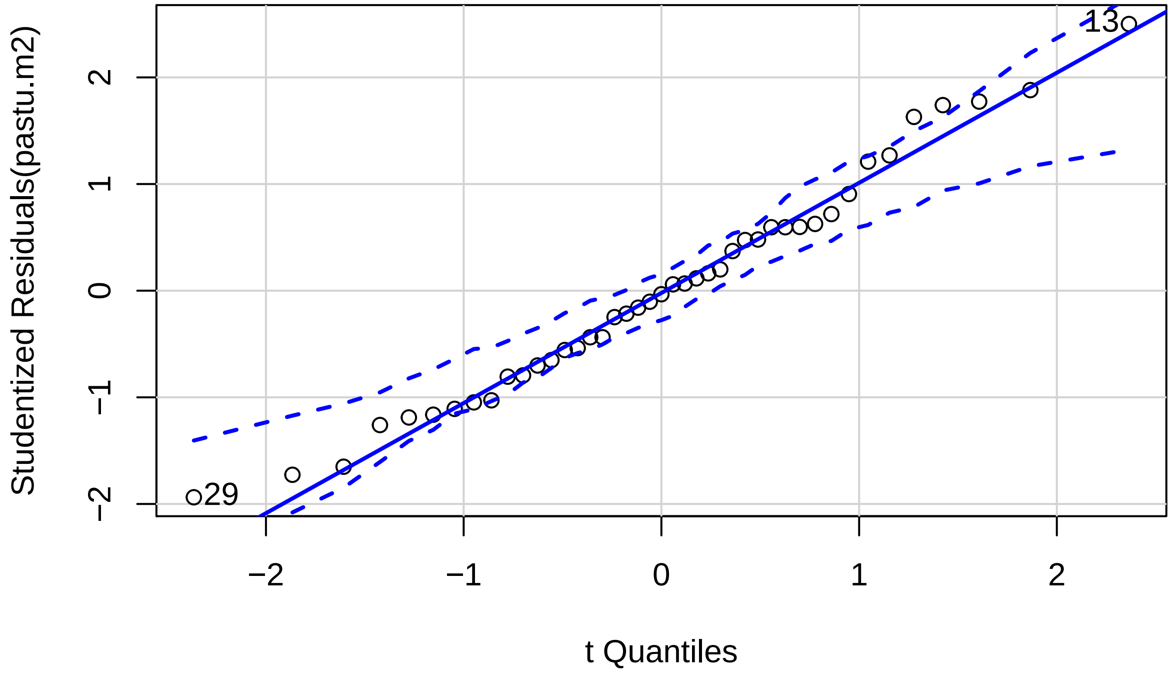


```
## [1] 13 29
```

```r
residualPlot(pastu.m2, quadratic = FALSE)
```

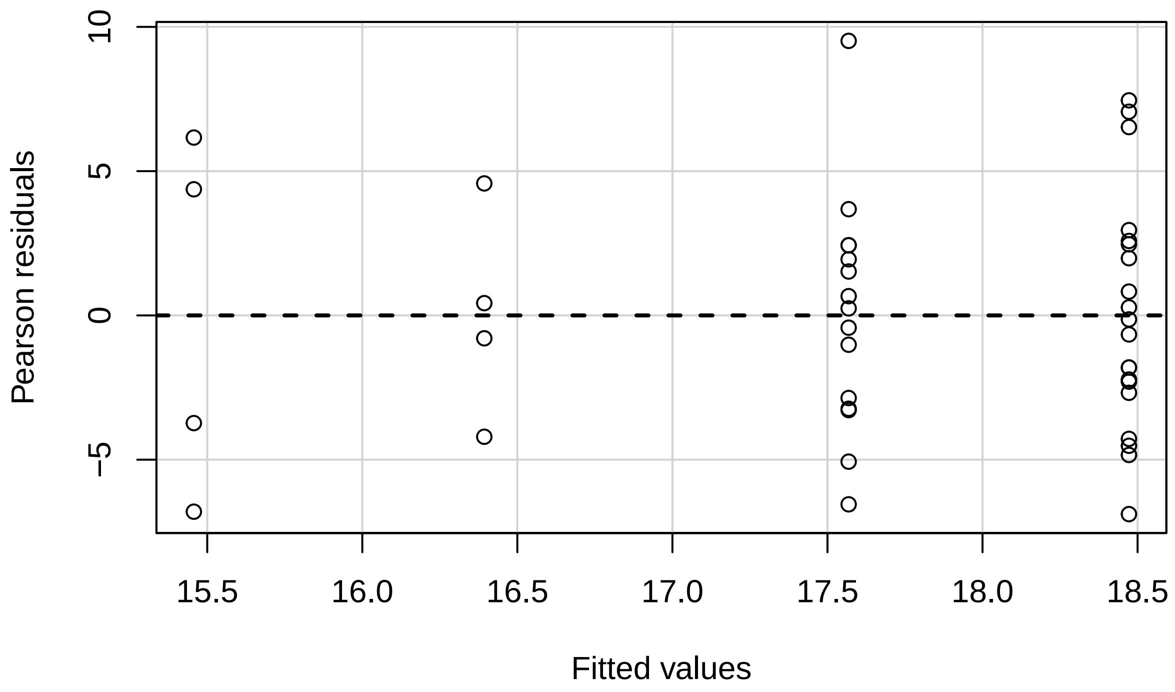


```r
#no interaction of pasture maturity and water access, note that here I am
#testing the hypothesis that only pre-optimum cutting point shouldn't have
#a negative impact on milk production when water access was limited
#that is why optimum and post-opt are combined.
```

THI

Hypothesis: High THI will decrease milk yield

Avg thi

```r
avgthi.m <- lm(avg.cow.d ~ avg_THI_roll, data = DF2)
summary(avgthi.m)
```

```
##
## Call:
## lm(formula = avg.cow.d ~ avg_THI_roll, data = DF2)
##
## Residuals:
##     Min      1Q  Median      3Q     Max
## -8.9165 -2.6201 -0.0027  2.4047  9.7141
##
## Coefficients:
##              Estimate Std. Error t value Pr(>|t|)
## (Intercept)  21.42613    8.34233   2.568   0.0134 *
## avg_THI_roll -0.05605    0.12355  -0.454   0.6521
## ---
## Signif. codes:  0 '***' 0.001 '**' 0.01 '*' 0.05 '.' 0.1 ' ' 1
##
## Residual standard error: 4.051 on 48 degrees of freedom
##   (2 observations deleted due to missingness)
## Multiple R-squared:  0.00427,    Adjusted R-squared:  -0.01647
## F-statistic: 0.2058 on 1 and 48 DF,  p-value: 0.6521
```

```r
qqPlot(avgthi.m)
```

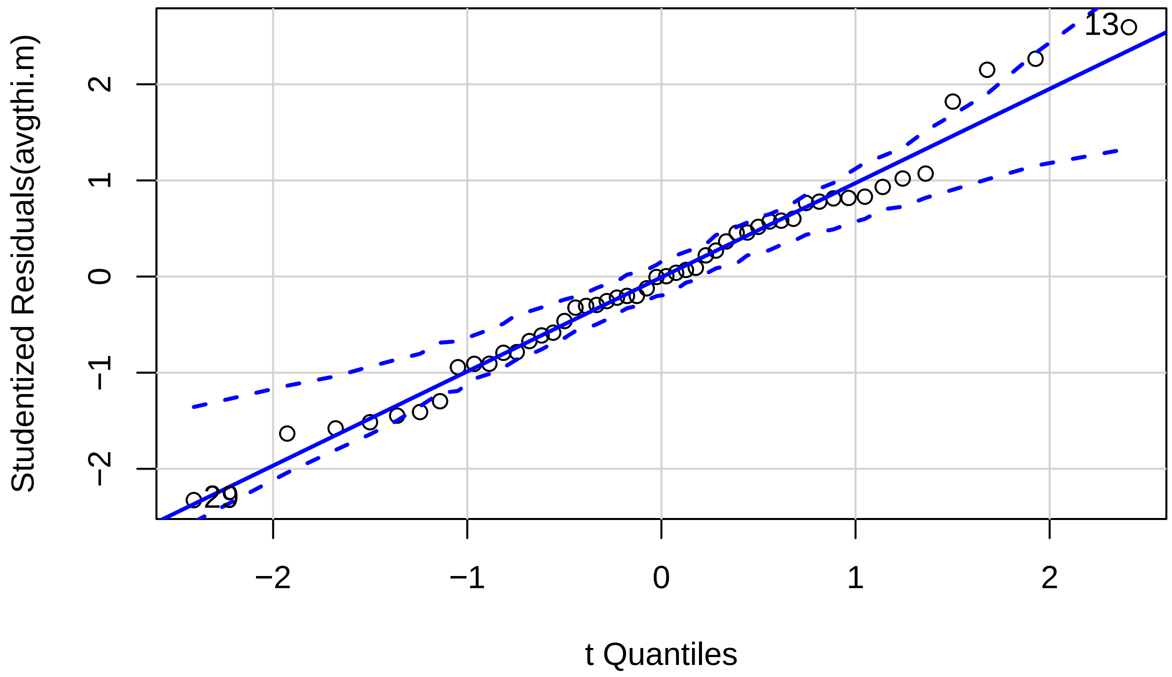


```
## [1] 13 29
```

```r
residualPlot(avgthi.m, quadratic = FALSE)
```

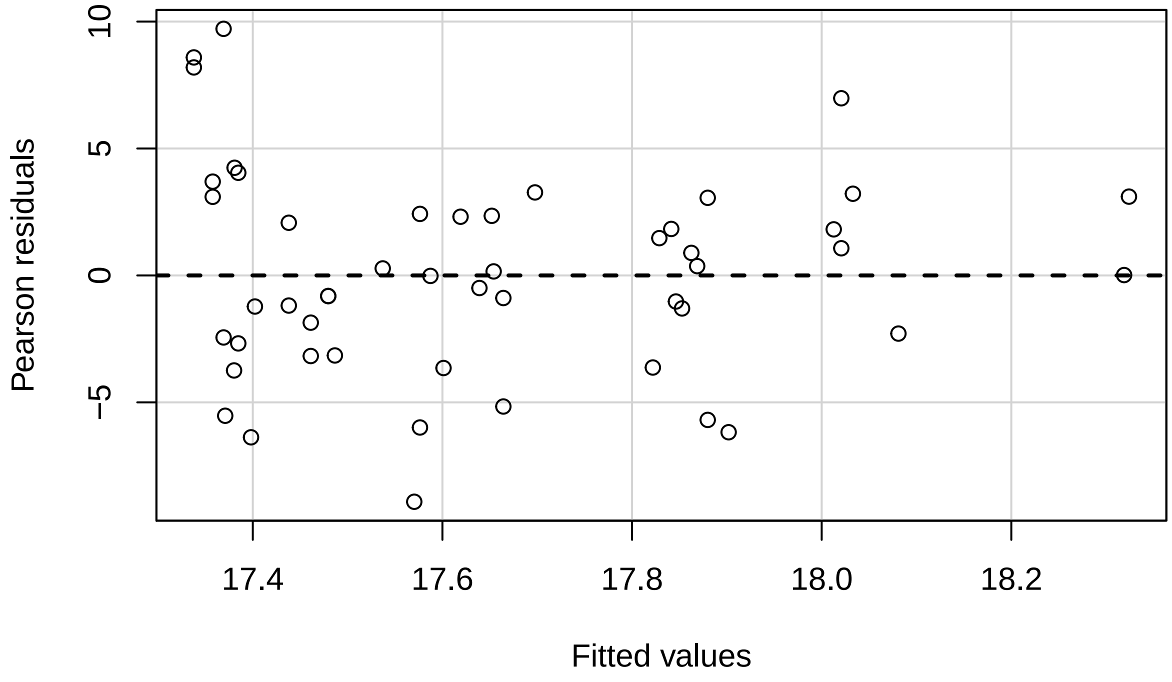

Sum THI74 hours

```r
thi74.m <- lm(avg.cow.d ~ sum_THI74_roll, data = DF2)
summary(thi74.m)
```

```
##
```

```
## Call:
## lm(formula = avg.cow.d ~ sum_THI74_roll, data = DF2)
##
## Residuals:
##     Min      1Q  Median      3Q     Max
## -8.9258 -3.0754  0.0558  2.3932  9.2963
##
## Coefficients:
##                Estimate Std. Error t value Pr(>|t|)
## (Intercept)    17.50325    0.81680  21.429   <2e-16 ***
## sum_THI74_roll  0.01092    0.04319   0.253    0.802
## ---
## Signif. codes:  0 '***' 0.001 '**' 0.01 '*' 0.05 '.' 0.1 ' ' 1
##
## Residual standard error: 4.057 on 48 degrees of freedom
##   (2 observations deleted due to missingness)
## Multiple R-squared:  0.001329,  Adjusted R-squared:  -0.01948
## F-statistic: 0.06389 on 1 and 48 DF,  p-value: 0.8015
```

```
qqPlot(thi74.m)
```

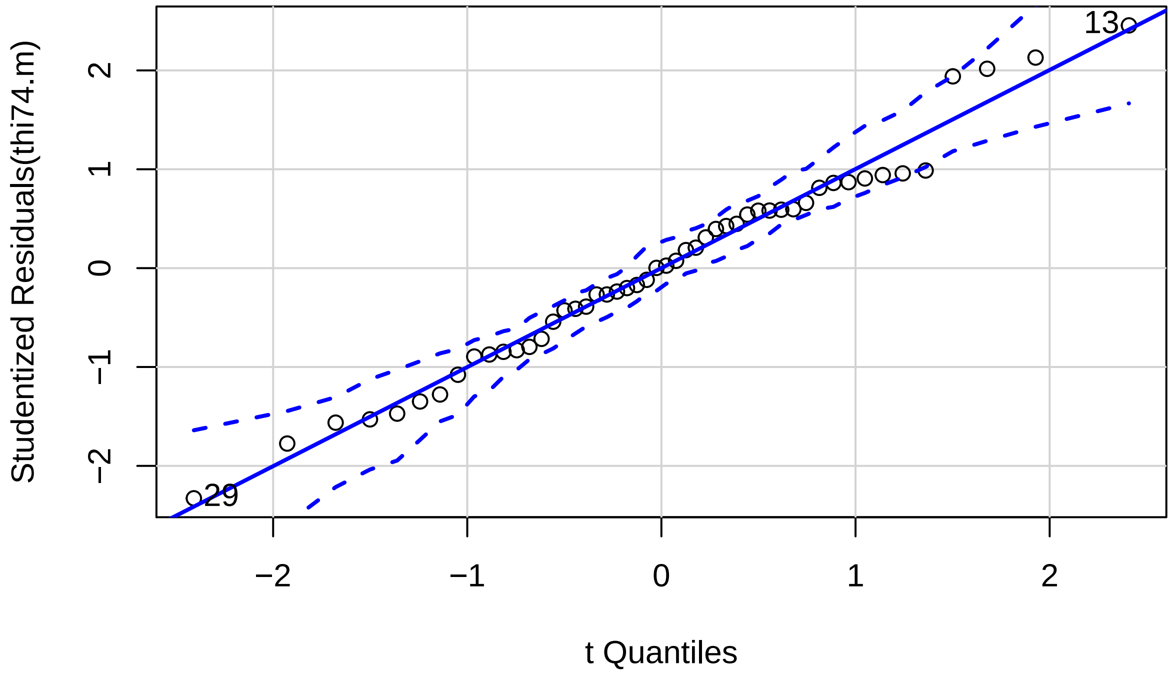


```
## [1] 13 29
```

```
residualPlot(thi74.m, quadratic = FALSE)
```

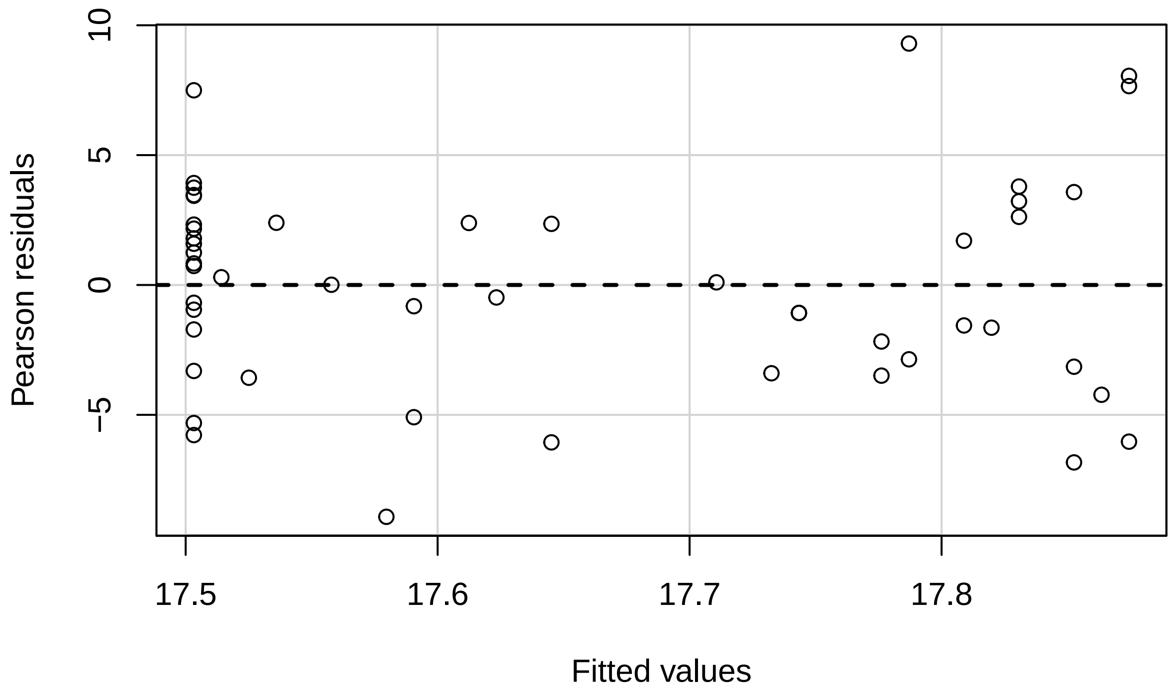

In summary THI is not associated with milk yield at the univariable level and will not be included in further models

Precipitation

before running the models let's graph the relantionship between precipitation, milk yield and type of water access

```
scatterplot(avg.cow.d ~ sum_precip_4d | as.factor(water.access), data = DF2)
```

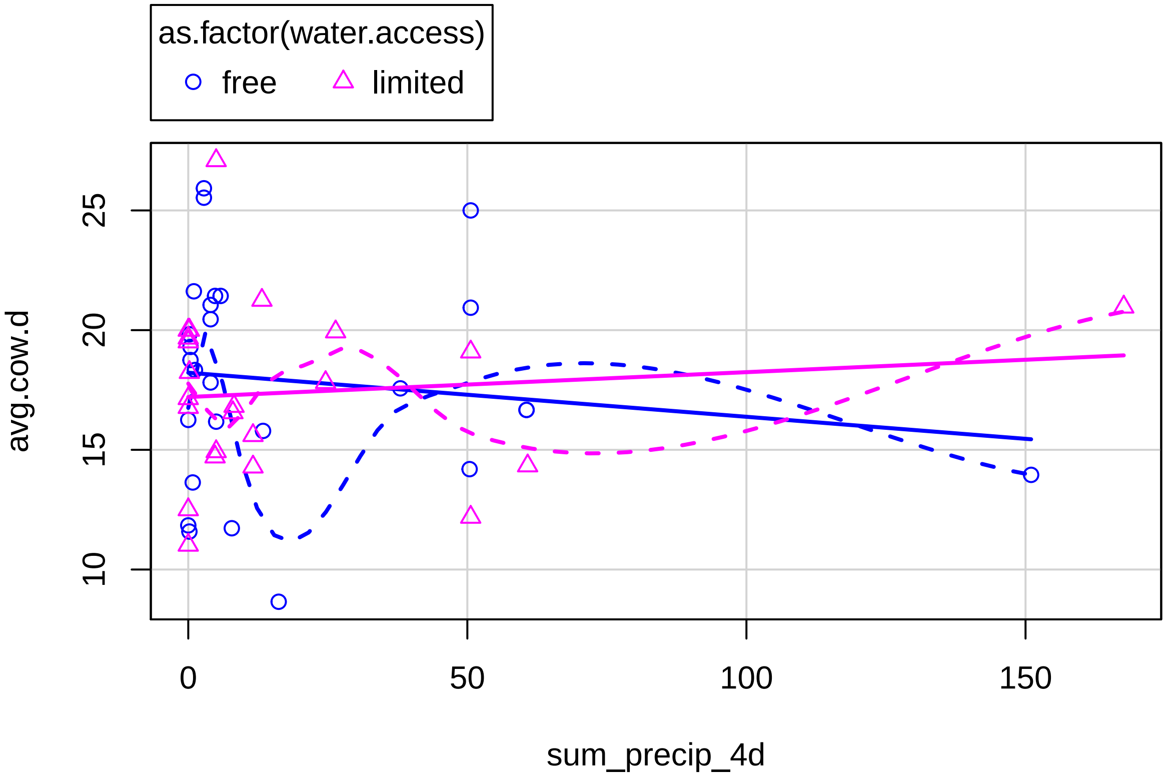

There is nothing here, let's model it anyway

```
precip.m <- lm(avg.cow.d ~ sum_precip_4d, data = DF2)
summary(precip.m)
```

```
##
## Call:
## lm(formula = avg.cow.d ~ sum_precip_4d, data = DF2)
##
## Residuals:
##     Min      1Q  Median      3Q     Max
## -9.0095 -2.9455  0.0448  2.2987  9.3777
##
## Coefficients:
##                Estimate Std. Error t value Pr(>|t|)
## (Intercept)   17.724512   0.661060  26.812   <2e-16 ***
## sum_precip_4d -0.003773   0.016656  -0.227    0.822
## ---
## Signif. codes:  0 '***' 0.001 '**' 0.01 '*' 0.05 '.' 0.1 ' ' 1
##
## Residual standard error: 4.058 on 48 degrees of freedom
##   (2 observations deleted due to missingness)
## Multiple R-squared:  0.001068,   Adjusted R-squared:  -0.01974
## F-statistic: 0.05132 on 1 and 48 DF,  p-value: 0.8217
```

```
qqPlot(precip.m)
```

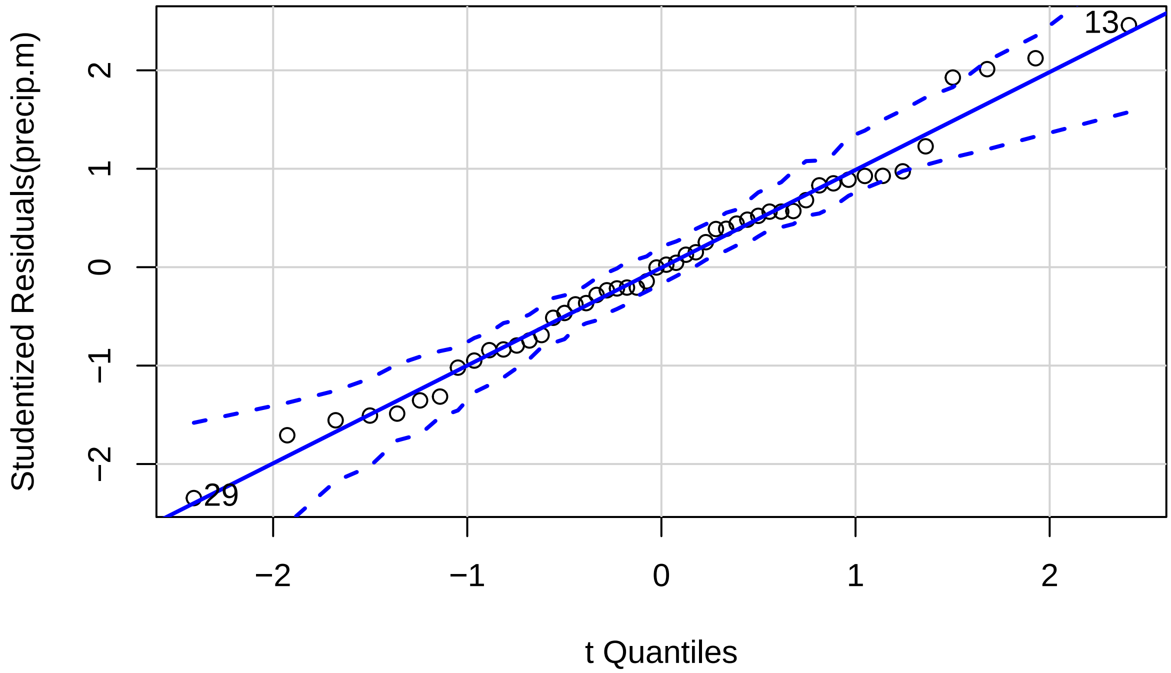


```
## [1] 13 29
```

```r
residualPlot(precip.m, quadratic = FALSE)
```

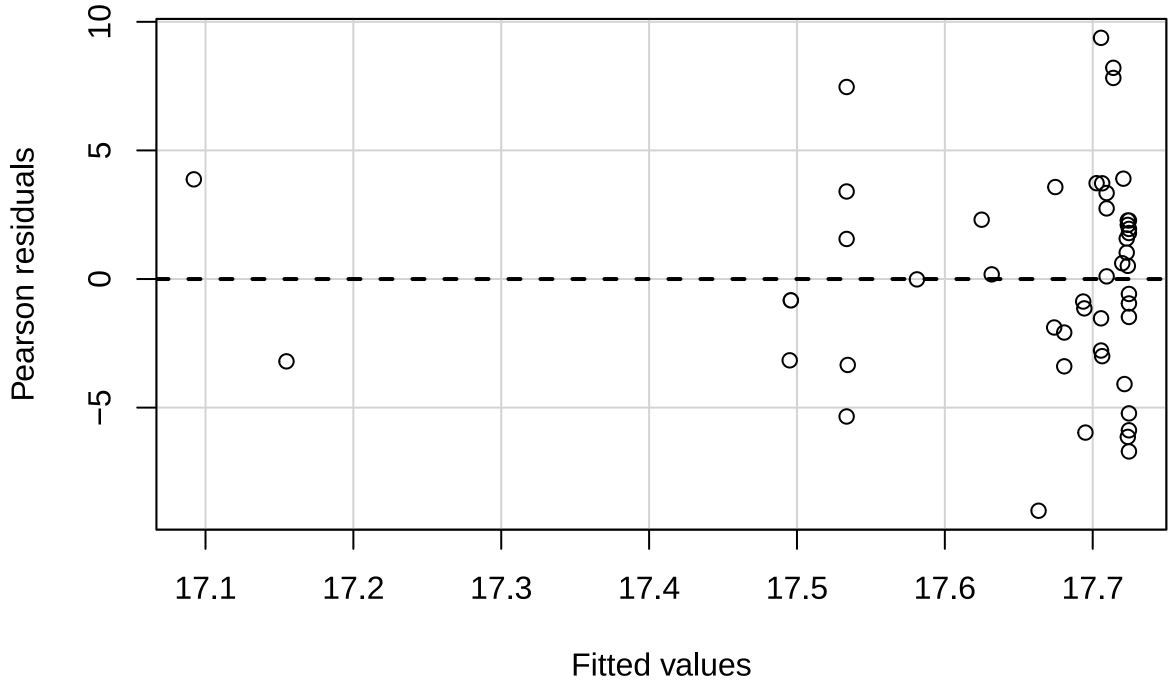

The variable precipitation will not be included in further models.

Visit order

Here we will double check if there was a visit order effect on water access type that may confound the results of water type access because if farms from one group were being assessed more in the summer than in the fall it may confound our results.

An elegant way of doing this would be to rank farms accordingly to their visit date and see if there is a rank

difference among them. For that we can use a wilcox rank sum test!

```
DF2$ranked_date <- rank(DF2$date, ties.method = "average")

wilcox.test(DF2$ranked_date ~ DF2$water.access, exact = F)
```

```
##
##  Wilcoxon rank sum test with continuity correction
##
## data:  DF2$ranked_date by DF2$water.access
## W = 318, p-value = 0.7278
## alternative hypothesis: true location shift is not equal to 0
```

In conclusion farms providing different types of water access for their herds were surveyed proportionately across the study period.

2.1.5 Hypothesis testing - Multivariable analysis

2.1.5.1 Selected variables

Breed (as categorical), amount of concentrate, silage area per cow, number of silage feedings per day.

2.1.5.1.1 Water access - unrestricted vs restricted

Categories: Restricted - cows did not have access to a water trough while on pasture and 2) Unrestricted - cows had free access to a water trough while on pasture.

```
m.water1 <- lm(avg.cow.d ~ mainbreed + concentrate.kg.cow.d
             + silage.area.cow + silage.freq.d + water.access, data = DF2)
summary(m.water1)
```

```
##
## Call:
## lm(formula = avg.cow.d ~ mainbreed + concentrate.kg.cow.d + silage.area.cow +
##     silage.freq.d + water.access, data = DF2)
##
## Residuals:
##     Min      1Q  Median      3Q     Max
## -4.7238 -1.5314  0.0967  1.9528  4.9918
##
## Coefficients:
##                         Estimate Std. Error t value Pr(>|t|)
## (Intercept)              12.2891     1.7881   6.873 2.49e-08 ***
## mainbreednon-holstein    -2.6288     0.8510  -3.089   0.0036 **
## concentrate.kg.cow.d      1.1311     0.2568   4.404 7.45e-05 ***
## silage.area.cow           4.4104     3.2811   1.344   0.1863
## silage.freq.d2            0.1200     0.9447   0.127   0.8996
## silage.freq.d3            0.1734     1.3155   0.132   0.8958
## water.accesslimited      -1.8683     0.8159  -2.290   0.0272 *
## ---
## Signif. codes:  0 '***' 0.001 '**' 0.01 '*' 0.05 '.' 0.1 ' ' 1
##
## Residual standard error: 2.615 on 41 degrees of freedom
##   (4 observations deleted due to missingness)
## Multiple R-squared:  0.5923, Adjusted R-squared:  0.5327
## F-statistic: 9.929 on 6 and 41 DF,  p-value: 9.277e-07
```

Freq of silage feeding per day seems not to be associated with milk yield anymore, let's remove it, check for indication of confounding (i.e. changes in the model estimates greater than 30%).

2.1.5.2 Final model

```
#FINAL MODEL
m.water2 <- lm(avg.cow.d ~ mainbreed + concentrate.kg.cow.d
              + silage.area.cow + water.access, data = DF2)
summary(m.water2)
```

```
##
## Call:
## lm(formula = avg.cow.d ~ mainbreed + concentrate.kg.cow.d + silage.area.cow +
##     water.access, data = DF2)
##
## Residuals:
##     Min      1Q  Median      3Q     Max
## -4.7569 -1.5798  0.1913  1.8731  5.0405
##
## Coefficients:
##                        Estimate Std. Error t value Pr(>|t|)
## (Intercept)             12.5349     1.7174   7.299 4.18e-09 ***
## mainbreednon-holstein   -2.7637     0.8274  -3.340  0.00171 **
## concentrate.kg.cow.d     1.1334     0.2498   4.538 4.36e-05 ***
## silage.area.cow          4.1012     2.9918   1.371  0.17738
## water.accesslimited     -1.7434     0.7643  -2.281  0.02743 *
## ---
## Signif. codes:  0 '***' 0.001 '**' 0.01 '*' 0.05 '.' 0.1 ' ' 1
##
## Residual standard error: 2.57 on 44 degrees of freedom
##   (3 observations deleted due to missingness)
## Multiple R-squared:  0.5851, Adjusted R-squared:  0.5474
## F-statistic: 15.51 on 4 and 44 DF,  p-value: 5.458e-08
```

```
emmeans::emmeans(m.water2, ~ mainbreed)
```

```
##  mainbreed      emmean        SE df lower.CL upper.CL
##  holstein     18.66596 0.5311855 44 17.59543 19.73650
##  non-holstein 15.90226 0.5785589 44 14.73625 17.06827
##
## Results are averaged over the levels of: water.access
## Confidence level used: 0.95
```

```
emmeans::emmeans(m.water2, ~ water.access)
```

```
##  water.access   emmean        SE df lower.CL upper.CL
##  free         18.15583 0.5003077 44 17.14752 19.16413
##  limited      16.41240 0.5624070 44 15.27894 17.54585
##
## Results are averaged over the levels of: mainbreed
## Confidence level used: 0.95
```

```
#removing the variable "silage.freq.d" doesn't change the r-sq, that is, this
#variable wasn't explaining much of the variation anyway.

# 95% CI
```

```r
confint(m.water2)
```

```
##                              2.5 %     97.5 %
## (Intercept)              9.0736338 15.9961446
## mainbreednon-holstein -4.4312807 -1.0961245
## concentrate.kg.cow.d   0.6300258  1.6367752
## silage.area.cow       -1.9283084 10.1307059
## water.accesslimited   -3.2836842 -0.2031777
```

```r
#Assessing model assumptions
vif(m.water2) #no indication of colinearity, all VIF values less than 2
```

```
##            mainbreed concentrate.kg.cow.d      silage.area.cow
##             1.265021             1.214825             1.114788
##         water.access
##             1.072000
```

```r
residualPlot(m.water2, quadratic = FALSE)
```

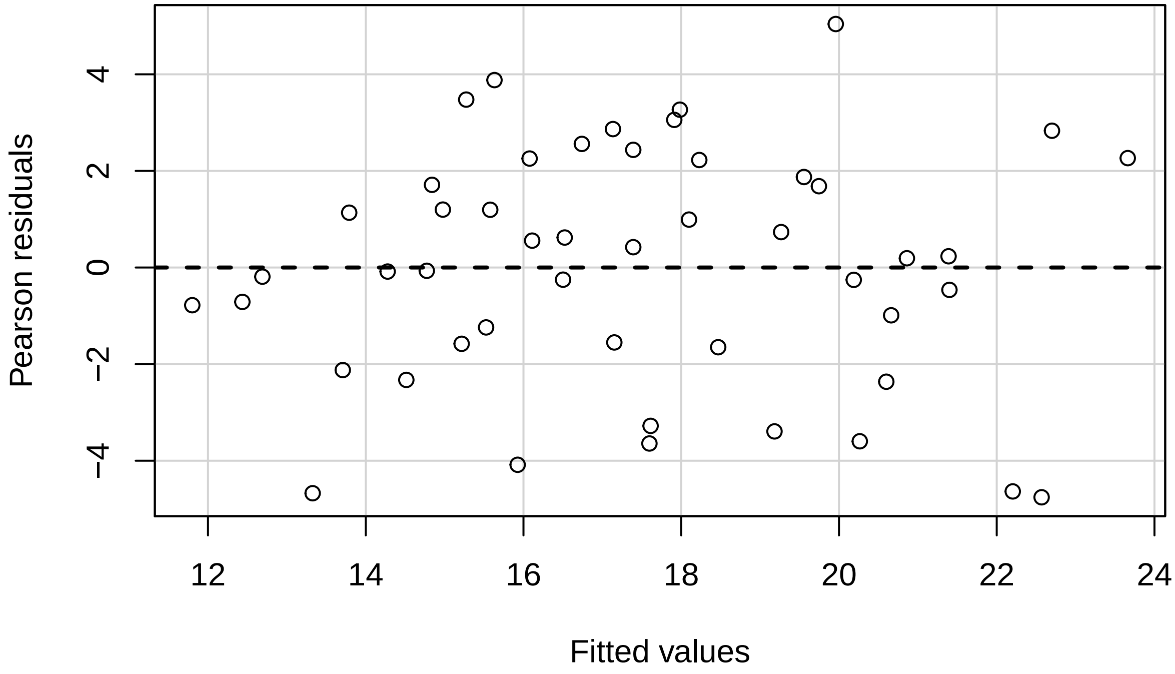


```r
#residuals are homoscesdastic
qqPlot(m.water2)
```

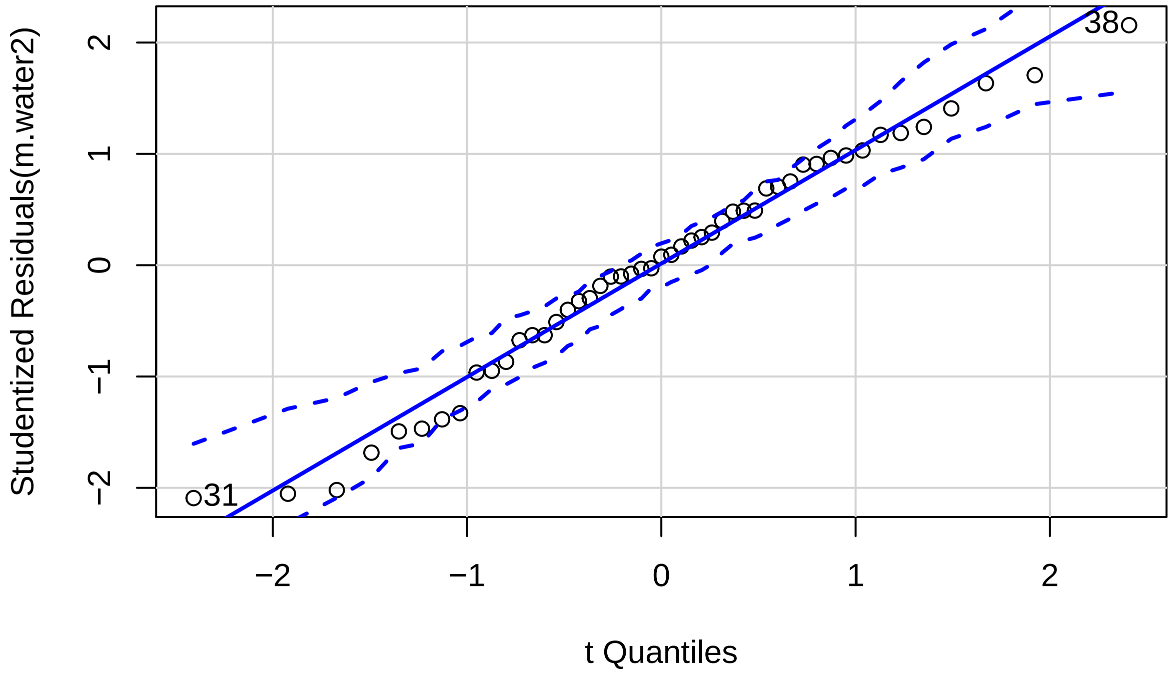


```
## [1] 31 38
```

```r
#and normally dist
avPlots(m.water2)
```

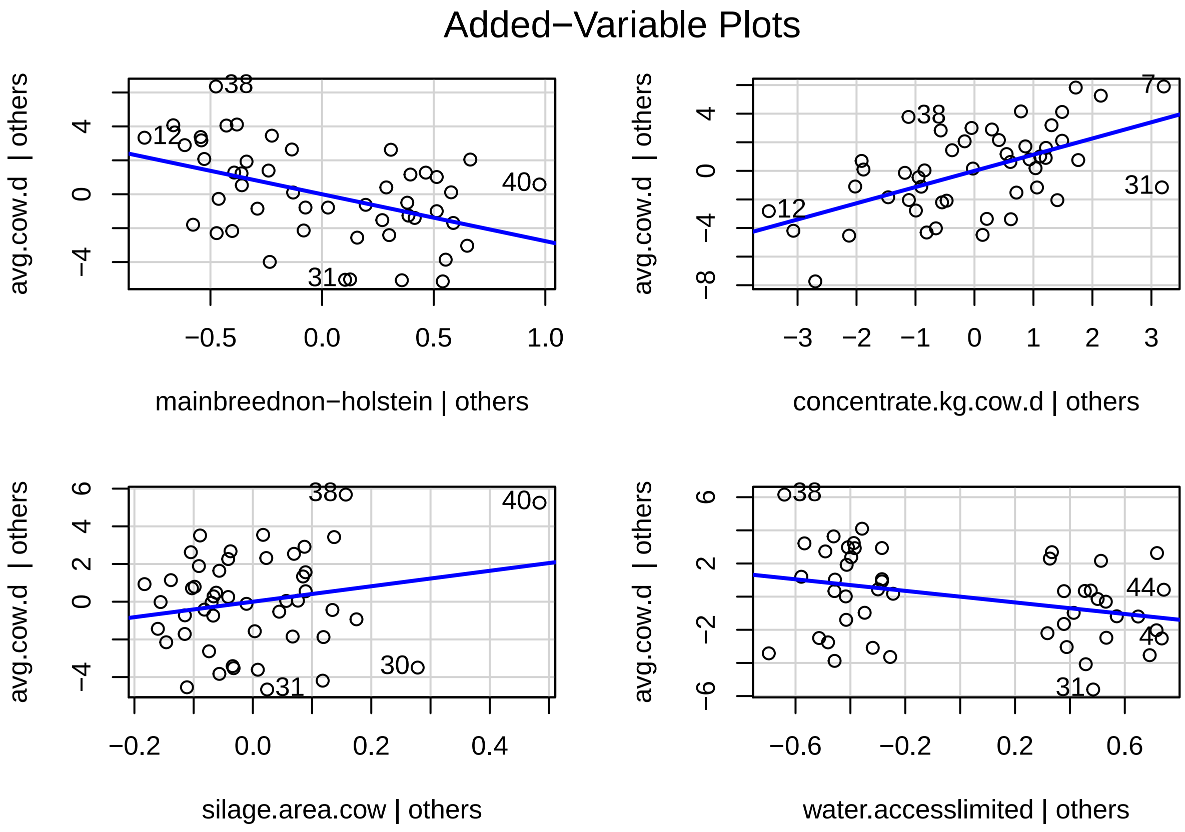


```r
#variables are somewhat linearly correlated
```

```r
#Now let's remove water from the model to see if it has any explanatory value
#in our model
m.water3 <- lm(avg.cow.d ~ mainbreed + concentrate.kg.cow.d
              + silage.area.cow, data = DF2)
summary(m.water3)
```

```
##
## Call:
## lm(formula = avg.cow.d ~ mainbreed + concentrate.kg.cow.d + silage.area.cow,
##     data = DF2)
##
## Residuals:
##     Min      1Q  Median      3Q     Max
## -5.6021 -2.2052  0.3324  2.2959  6.1576
##
## Coefficients:
##                      Estimate Std. Error t value Pr(>|t|)
## (Intercept)           11.7077     1.7554   6.670 3.15e-08 ***
## mainbreednon-holstein  -2.4520     0.8533  -2.873  0.00618 **
## concentrate.kg.cow.d    1.1753     0.2605   4.512 4.57e-05 ***
## silage.area.cow         2.9360     3.0825   0.952  0.34594
## ---
## Signif. codes:  0 '***' 0.001 '**' 0.01 '*' 0.05 '.' 0.1 ' ' 1
##
## Residual standard error: 2.687 on 45 degrees of freedom
##   (3 observations deleted due to missingness)
## Multiple R-squared:  0.536,  Adjusted R-squared:  0.5051
## F-statistic: 17.33 on 3 and 45 DF,  p-value: 1.271e-07
```

```r
anova(m.water3, m.water2)
```

```
## Analysis of Variance Table
##
## Model 1: avg.cow.d ~ mainbreed + concentrate.kg.cow.d + silage.area.cow
## Model 2: avg.cow.d ~ mainbreed + concentrate.kg.cow.d + silage.area.cow +
##     water.access
##   Res.Df    RSS Df Sum of Sq     F  Pr(>F)
## 1     45 324.99
## 2     44 290.62  1    34.372 5.204 0.02743 *
## ---
## Signif. codes:  0 '***' 0.001 '**' 0.01 '*' 0.05 '.' 0.1 ' ' 1
```

```r
#The anova shows that removing the water access variable, increases
#significatively the residuals sum of square (RSS).
# i.e. the variable water access has explanatory value.
```

3 Conclusion

Water access is significantly associated with milk production.
